# Single-cell Analysis Technologies for Immuno-oncology Research: from Mechanistic Delineation to Biomarker Discovery

**DOI:** 10.1016/j.gpb.2021.02.004

**Published:** 2021-05-14

**Authors:** Zhiliang Bai, Graham Su, Rong Fan

**Affiliations:** 1 Department of Biomedical Engineering, Yale University, New Haven, CT 06511, USA; 2 State Key Laboratory of Precision Measurement Technology and Instrument, Tianjin University, Tianjin 300072, China; 3 Yale Stem Cell Center and Yale Cancer Center, Yale School of Medicine, New Haven, CT 06511, USA; 4 Human and Translational Immunology, Yale School of Medicine, New Haven, CT 06511, USA

**Keywords:** Single-cell analysis, Immunotherapy, Biomarker discovery, Checkpoint blockade, CAR-T

## Abstract

The successes with immune **checkpoint blockade** (ICB) and chimeric antigen receptor (CAR)-T-cell therapy in treating multiple cancer types have established **immunotherapy** as a powerful curative option for patients with advanced cancers. Unfortunately, many patients do not derive benefit or long-term responses, highlighting a pressing need to perform complete investigation of the underlying mechanisms and the immunotherapy-induced tumor regression or rejection. In recent years, a large number of single-cell technologies have leveraged advances in characterizing immune system, profiling tumor microenvironment, and identifying cellular heterogeneity, which establish the foundations for lifting the veil on the comprehensive crosstalk between cancer and immune system during immunotherapies. In this review, we introduce the applications of the most widely used single-cell technologies in furthering our understanding of immunotherapies in terms of underlying mechanisms and their association with therapeutic outcomes. We also discuss how **single-cell analyses** help to deliver new insights into **biomarker discovery** to predict patient response rate, monitor acquired resistance, and support prophylactic strategy development for toxicity management. Finally, we provide an overview of applying cutting-edge single-cell spatial-omics to point out the heterogeneity of tumor–immune interactions at higher level that can ultimately guide to the rational design of next-generation immunotherapies.

## Introduction

Hailed as one of the most promising cancer treatments today, immunotherapy has become a new therapeutic option for patients and has demonstrated impressive clinical benefit in treatment of many types of malignancies. Instead of directly targeting the cancer, the goal of immunotherapy is to deliberately and specifically enhance our immune system to fight back, which induces longer-lasting durable responses in a proportion of patients. Currently, there are several types of immunotherapies with the main ones being cancer vaccines, oncolytic virus treatment, immune checkpoint blockade (ICB), and adoptive cellular therapy (ACT). However, despite the U.S. Food and Drug Administration (FDA)-approved cancer vaccine for the treatment of prostate cancer (sipuleucel-T) [1] and oncolytic virus used for patients with recurrent melanoma (talimogene laherparepvec; T-VEC) [2], the overall clinical trials for these two immunotherapies have turned out to be less than promising.

Following these, we move into ICB, which has been showing increasingly significant clinical response for multiple malignancies. Immune checkpoints are the co-stimulatory and inhibitory signals that regulate and protect against uncontrolled immune response that may cause undesired inflammation or damage to the host [3]. Cytotoxic T-lymphocyte-associated protein 4 (CTLA-4), which was first discovered in 1987 [4], is one such immune checkpoint protein that predominantly regulates naïve effector T-cell activation in lymphocytes. Also highly characterized are programmed cell death protein 1 (PD-1) and programmed death ligand 1 (PD-L1), which make up the second category of immune checkpoint therapies. They differ from CTLA-4 in that they regulate primed T-cell activity in peripheral tissue and the tumor microenvironment (TME). Ipilimumab is the first ICB therapy that was approved by FDA in 2011 and it inhibits CTLA-4 in unresectable or metastatic melanoma [5]. Pembrolizumab, the first PD-1 checkpoint inhibitor, was approved in September 2014 for the treatment of patients with metastatic melanoma and shortly after, Nivolumab, another PD-1 checkpoint inhibitor, was approved in December 2014 and is currently used in treatment of many malignancies including melanoma, lung cancer, kidney cancer, and bladder cancer [6].

ACT has also yielded remarkable clinical responses, especially in the treatment of patients with hematologic cancers. The main idea behind adoptive cellular immunotherapy is to activate the patient’s own immune cells *ex vivo* to better target and destroy the cancer cells after being reintroduced into the patient. The first successful attempt was utilizing tumor-infiltrating lymphocytes (TILs) through isolating lymphocytes from tumor biopsies in melanoma [7], yet it is difficult to expand to a sufficient amount for other types of cancer. Following this, patient T-cells were isolated *ex vivo* and transfected with a chimeric antigen receptor (CAR) that consists of a fragment of an antibody specific to a particular antigen such as CD19. This CAR-T cell therapy showed remarkable success in patients with B cell acute lymphoblastic leukemia (ALL), but treating patients with solid tumors has been proving difficult so far [8]. Since the earliest commercially available CAR-T product approved in 2017 (Kymriah from Novartis Pharmaceuticals Corp.), FDA has granted approval for four CAR-T-cell therapies until very recently. Lastly, a third type of ACT is T-cell receptor (TCR)-T-cell therapy, which shares a similar idea as CAR-T-cell therapy but instead the patient’s T-cells are transfected with a TCR that is specific to a tumor antigen.

Although the success stories of immunotherapy are promising, further improvements are still urgently needed. A huge problem remaining for ICB and ACT is why some patients respond extremely well to the treatment while others show no response, acquire induced resistance, or experience harmful side effects. Not only there are immunotherapy-specific side effects such as cytokine release syndrome (CRS) and neurotoxicity in CAR-T treatment, but also there are shared issues common to all immunotherapies, such as unwanted off-target effects, unpredictable efficacy, and difficulty of identifying biomarkers [9], [10].

Understanding how to elevate the response rates to a specific type of immunotherapy or combination therapy is indispensable for improving efficacy and also controlling adverse effects, which relies on an accurate dissection of the cellular composition of the TME and the interplay between all kinds of cells before and after immunotherapy. With the advent of single-cell technologies together with next-generation sequencing (NGS) technologies, now it is possible to address these issues in a more comprehensive, informative, and robust manner. Compared with bulk-cell level characterization, single-cell technologies can provide unique information of the cell-to-cell heterogeneity in human cancer and investigate the roles it plays in response to treatments at various levels of the central dogma of molecular biology. Over the last decade, advances in accuracy, throughput, computational analysis, and cost of single-cell technologies revolutionize the paradigm we study both tumor and immune system. Now it is feasible for researchers to select suitable single-cell method according to the tumor type, number of available cells, molecular layer of interest, technical properties, and cost considerations.

Many review articles have been published to summarize how single-cell technologies were developed to study proteomics, transcriptomics, epigenomics and multi-omics [11], [12], [13], [14], [15]. In this review, we mainly discussed how the use of these technologies has yielded great insights into the ability to understand the mechanisms, predict patient response rate, monitor acquired resistance, and manage anticipated toxicity in immuno-oncology studies. Additionally, we prepared a systematic summary of the most widely used single-cell methods and supplied it as supplement to provide convenience for readers who are not familiar with these techniques (File S1).

## Delineating the mechanisms underlying immunotherapies

By boosting or restoring the immune system functions, immunotherapies can marshal the specificity of adaptive immune response to destroy cancer cells and shift the equilibrium back in favor of immune surveillance. Antibodies targeting PD-1, PD-L1, or CTLA-4 have demonstrated durable responses in the treatment of a variety of solid tumor malignancies. Meanwhile, anti-CD19 CAR-T cells have induced complete remission in over 90% of cases in relapsed or refractory ALL [16]. However, we still lack a complete understanding of the fundamental mechanisms that underly the immunotherapy-induced tumor regression or rejection, which is necessary for the improvement of current therapies and for the rational design of combination therapy approaches. With continuing advances in high-throughput single-cell technologies, we can profile the host immune response and elucidate the tumor intrinsic properties that are related to the therapeutic outcomes. In this section, we present studies that have used single-cell technologies to understand the characteristics of TILs during ICB, to explore the mechanisms involved in remodeling the TME after ICB, as well as to evaluate the properties of cellular products pre- or post-infusion. Table 1 lists the studies that use single-cell technologies to delineate the mechanisms of immunotherapy.

**Table 1:** **Summary of the studies employing single-cell technologies to delineate the mechanisms of immunotherapy**

*Note*: “+” means combined immunotherapy; CyTOF, mass cytometry; scRNA-seq, single-cell RNA-sequencing; CTLA-4, cytotoxic T-lymphocyte-associated protein 4; PD-1, programmed cell death protein 1; TIL, tumor infiltrating lymphocyte; TCR-seq, T cell receptor sequencing; scTCR-seq, single-cell T cell receptor sequencing; GITR, glucocorticoid-induced tumor necrosis factor receptor-related protein; HLA, human leukocyte antigen; IsoCode, single cell antibody coated chip commercialized by Isoplexis; ICB, immune checkpoint blockade; CAR-T, chimeric antigen receptor T; BCMA, B cell maturation antigen; TACI, transmembrane activator, calcium modulator, and cyclophilin ligand interactor; NHL, non-Hodgkin lymphoma; ACT, adoptive cell therapy; TCR-T, T cell receptor-engineered T; ALL, acute lymphoblastic leukemia; NY-ESO-1, New York esophageal squamous cell carcinoma 1; GM-CSF, granulocyte–macrophage colony-stimulating factor; N/A, not applicable.

### Characterization of tumor-infiltrating T cells during ICB

Accumulating evidence indicates that the location, density, and functional orientation of tumor-infiltrating immune cells play a critical role in the clinical outcome of cancer patients [17], [18]. Major components of the infiltrated immune populations are CD8^+^ and CD4^+^ T cells that can essentially contribute to tumor elimination. Thus, tumor-infiltrating T cells have been the early focus for understanding the mechanisms of ICB, since they are direct targets of anti-PD-1 or anti-CTLA-4.

**Figure 1:**
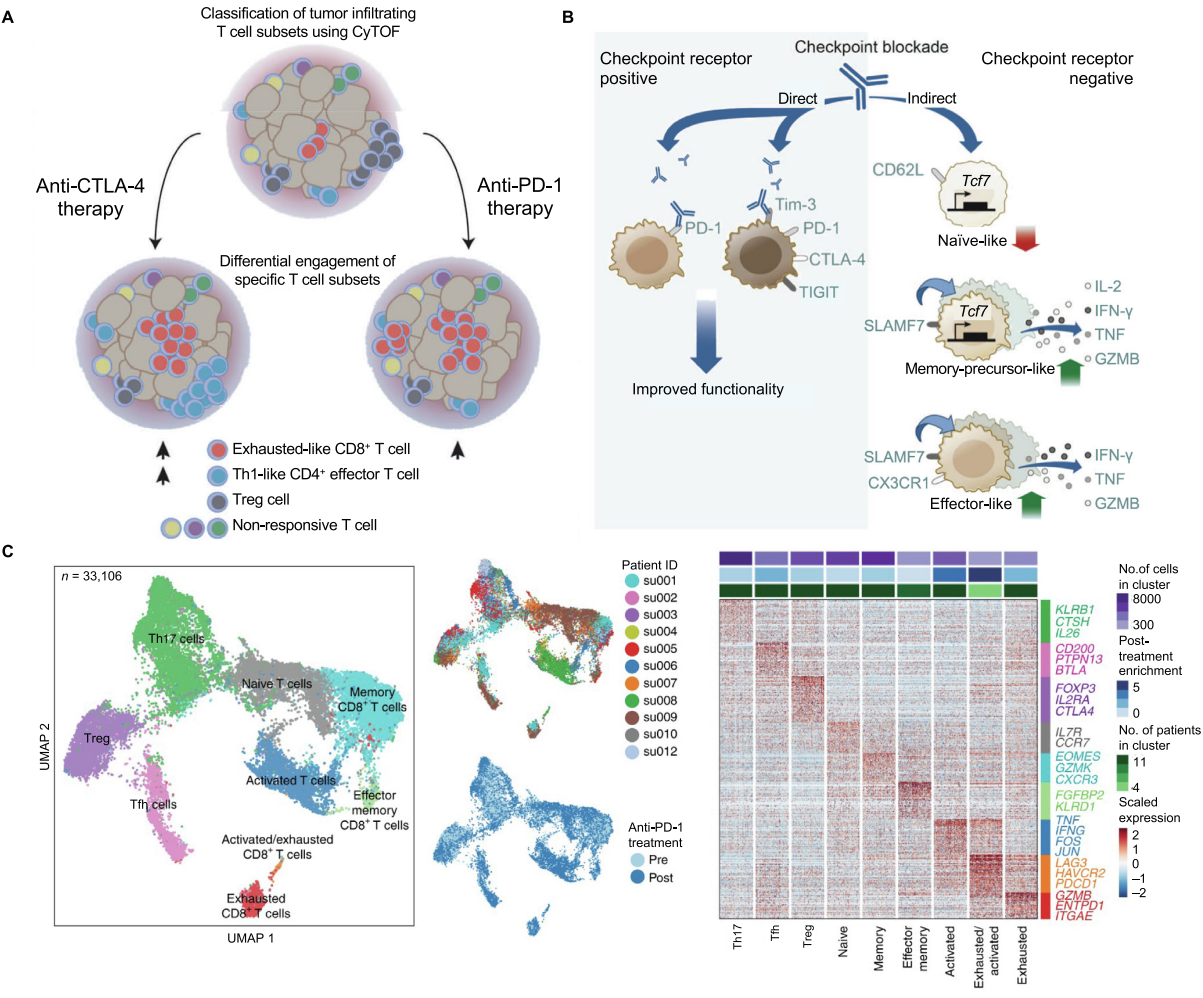
**Representative studies employing single-cell technologies to characterize TILs during ICB to investigate the response-driven molecular determinants** **A.** Using CyTOF to compare the cellular mechanisms underlying anti-PD-1 and anti-CTLA-4-induced anti-tumor immune response [19]. **B.** Using both bulk RNA-seq and scRNA-seq to profile the dynamic changes in PD-1^−^CD8^+^ tumor-infiltrating T cells induced by ICB. Three subsets with features of naive, memory-precursor, or effector CD8^+^ T cells have been identified [22]. **C.** Using scRNA-seq and TCR sequencing to investigate the clonal replacement of tumor-specific T cells following PD-1 blockade [23]. On the left: UMAP showing clusters of tumor-infiltrating T cells present in basal or squamous cell carcinoma samples before and after PD-1 blockade; on the right: heatmap displaying DEGs between cells belonging to different T cell subsets. DEGs that are associated with different T cell clusters are highlighted. Panel A is adapted from [19] with permission. Panel B is adapted from [22] with permission. Panel C is adapted from [23] with permission. TIL, tumor-infiltrating lymphocyte; ICB, immune checkpoint blockade; CyTOF, mass cytometry; scRNA-seq, single-cell RNA-sequencing; TCR, T cell receptor; UMAP, uniform manifold approximation and projection; DEG, differentially expressed gene; Tfh, follicular helper T cell; Treg, regulatory T cell.

In 2017, Wei et al. utilized mass cytometry (also known as cytometry by time-of-flight; CyTOF) to comprehensively profile the immune infiltrates in human melanoma and murine tumor models following two different ICB therapies [19] (Figure 1A). They have first verified that the phenotypes of infiltrating T cells and mechanisms of ICB are indeed tumor-type-independent, and anti-CTLA-4-induced and anti-PD-1-induced anti-tumor responses are driven by distinct cellular mechanisms. Specifically, both ICB therapies only target a subset of tumor-infiltrating T cell populations, inducing the expansion of exhausted-like CD8^+^ T cells. In addition to that, CTLA-4 blockade also induces expansion of inducible T cell costimulator (ICOS)^+^ Th1-like CD4^+^ effector compartment, although the definitive source (anatomical and temporal) and precise function of this expansion remain unclear.

Additional studies have continued to uncover the previously-unknown heterogeneity of CD8^+^ TILs in the ICB immunotherapy. To better understand the basis for T cell dysfunction in cancer and the mechanism through which anti-PD-1 therapy reinvigorates T cell function, Miller et al. applied single-cell RNA-sequencing (scRNA-seq) to define the heterogeneity in exhausted CD8^+^ T cells from patients chronically infected with lymphocytic choriomeningitis virus [20].They have found two major subsets of exhausted T cells in the TME: a progenitor population with greater polyfunctionality and the ability to persist in the absence of antigen, and a terminally-exhausted population with superior cytotoxicity but reduced long-term survival. Notably, the progenitor subset of exhausted T cells can mediate superior tumor control and respond to PD-1 blockade, suggesting that the efforts to increase their frequency may represent an important therapeutic strategy. Another study performed by Fehlings et al. focused on neoantigen-specific CD8^+^ T cells, and using CyTOF, they tested 81 candidate antigens across tissues from mice bearing progressive sarcomas [21]. It is found that neoantigen-specific TILs have a higher frequency of cells co-expressing PD-1 and Tim-3, and there is an increase in the magnitude of mLama4-specific and mAlg8-specific TILs following anti-CTLA-4. More importantly, their results also show that antigen-specific T cells in response to anti-CTLA-4 or anti-PD-1 immunotherapies acquire a similar novel phenotypic diversity, providing insights into the nature of neoantigen-specific T cells and the effects of checkpoint blockade immunotherapy.

Understanding which subsets of CD8^+^ TILs give rise to the effector response upon checkpoint blockade could enable improved strategies for harnessing the CD8^+^ T cell response in fighting cancer. Kurtulus et al. used both bulk and single-cell RNA-seq to examine the dynamics of the effector response of CD8^+^ TILs [22] (Figure 1B). Their results show that infiltrating T cells lack the expression of PD-1 and other checkpoint receptors are then recruited during the therapy. Specifically, three subsets of PD-1^−^CD8^+^ TILs with features of naive, memory-precursor, or effector cells have been identified, and the expansion of the memory-precursor and effector-like PD-1^−^CD8^+^ TIL subsets with concomitant decrease in the naive-like subset is induced during ICB. In addition, their data show that in the absence of *Tcf7*/*Tcf1*, the memory-precursor-like subset is compromised, leading to failed immunotherapies. These results have important clinical implications for monitoring therapeutic response, identifying targets that can be modulated in T cells, and ensuring sustained and durable responses.

To elucidate whether the T cell response to ICB relies on reinvigorating pre-existing TILs or on recruiting novel T cells, very recently, Yost et al. combined scRNA-seq and TCR sequencing to track TCR clones and transcriptional phenotypes of 79,046 single cells derived from patients with basal or squamous cell carcinoma before and after anti-PD-1 therapy [23] (Figure 1C). Through profiling TILs, they find that the proportion of exhausted CD8^+^ TILs increases after PD-1 blockade and these cells express gene signatures of chronic activation, T cell dysfunction, and tumor reactivity. Then, the lineage relationships between T cell phenotypes and clonotypes have been analyzed and the results suggest that clonally expanded TILs are highly correlated in cellular phenotype and that PD-1 blockade does not promote phenotypic instability within a clone. Following treatment, Yost et al. have investigated how clone abundance changes globally by comparing pre- and post-treatment frequencies of each clone, and have identified that post-treatment exhausted clones are significantly enriched for novel clonotypes. These results reveal insights into the clonal T cell response to checkpoint blockade and demonstrate that the response derives from a distinct repertoire of T cell clones that may have just recently entered the tumor.

Unmasking the molecular pathways that drive durable antitumor responses is also crucial to the development of rational approaches to optimizing ICB. By employing single-cell TCR sequencing and scRNA-seq, Wang et al. [24] sought to establish an experimental approach to identify the pathways of combination therapy of anti-glucocorticoid-induced tumor necrosis factor receptor-related protein (GITR) [25] and anti-PD-1 antibodies. They find that the combination synergistically enhances the effector function of expanded CD8^+^ T cells by restoring the balance of two key homeostatic regulators, CD226 and T cell immunoreceptor with Ig and ITIM domains (TIGIT), and results in robust survival benefit. Gene pathway analysis reveals that the combination treatment also integrates the molecular pathways modulated by anti-GITR or anti-PD-1 monotherapies. Furthermore, the activation module has significantly higher scores in the combination treatment than monotherapy treatment groups. Accordingly, CD226 is confirmed as the most up-regulated gene upon combination treatment.

### Profiling the cellular reprogramming of TME in ICB

While the aforementioned studies focused on intratumoral T cells, several groups have described the use of single-cell technologies to understand how immune checkpoint blockers engage complex TME and which mechanisms are associated with treatment success.

**Figure 2:**
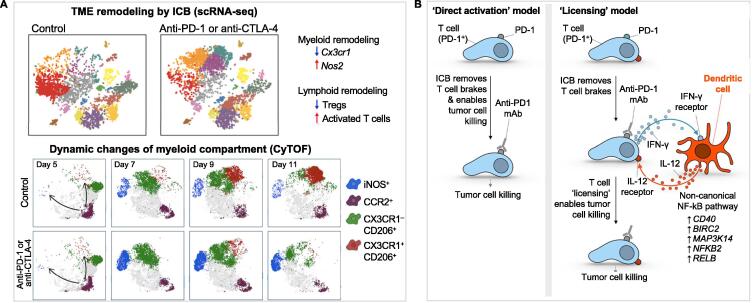
**Representative studies employing single-cell technologies to profile the cellular reprogramming of TME during ICB** **A.** Combining scRNA-seq and CyTOF to characterize all hematopoietic cells from syngeneic mouse tumors during unrestrained tumor growth or effective checkpoint therapy. TME remodeling and dynamic changes of myeloid compartment are observed over time during the ICB therapy [27]. **B.** combining intravital real-time imaging and scRNA-seq to show that successful anti-PD-1 cancer immunotherapy requires T cell–dendritic cell crosstalk involving cytokines IFN-γ and IL-12 [29]. Panel A is adapted from [27] with permission. Panel B is adapted from [29] with permission. mAb, monoclonal antibody; TME, tumor microenvironment.

Successful ICB therapy engages multiple classes of immune networks in the TME to defend the host against tumor development, and high dimensional single-cell profiling approaches have been used to provide insights into transcriptional, molecular, and functional changes that are involved in the checkpoint inhibition. In a hepatocellular carcinoma study, Chew et al. used CyTOF to dissect the precise dynamics between the TME, non-TME, and the peripheral blood cells [26]. It is found that a specific subset of PD-1^+^ tissue resident memory CD8^+^ T cells plays the predominant role in the response to anti-PD-1 treatment and is significantly reduced in number along with the tumor progression. Furthermore, T-bet is identified as a key transcription factor whose expression is negatively correlated with PD-1 expression on memory CD8^+^ T cells, and the PD-1:T-bet ratio increases upon exposure to tumor antigens. This study also confirms the existence of an immunosuppressive gradient that immune cell subsets become progressively suppressive as they traverse the non-TME to TME. In another study, Gubin et al. employed two different high-dimensional analysis approaches, scRNA-seq and CyTOF, to characterize all hematopoietic cells from syngeneic mouse tumors during unrestrained tumor growth or effective checkpoint therapy [27] (Figure 2A). They have observed multiple subpopulations of monocytes and macrophages that change over time during ICB, strongly supporting the argument for the need to consider engaging both innate and adaptive immunity to improve the efficacy of cancer immunotherapy. Very recently, scRNA-seq analyses performed by Wisdom et al. have revealed that despite the immunosuppressive TME of untreated primary sarcomas, PD-1 blockade and radiation therapy successfully repolarize myeloid cells in primary tumors, with the dominant changes being activation of type I and II interferon response pathways [28].

Although immunotherapies have emerged as a promising approach to mediate durable cancer regression, partial responses across different types of cancers emphasize the importance of characterizing intertumoral and intratumoral heterogeneities to achieve better clinical results. To fully investigate the immune response-related genes and regulatory pathways and to evaluate their potential impact on the efficacy of immunotherapies, single-cell technologies have become a rapidly evolving method. Combining intravital real-time imaging with scRNA-seq, Garris et al. have shown that successful antitumor responses require crosstalk between a subset of tumor-infiltrating dendritic cells (DCs) in anti-PD-1 immunotherapy [29] (Figure 2B). Principally, they have found that anti-PD-1 directly induces interferon-γ (IFN-γ) production by activated T cells, but indirectly induces interleukin-12 (IL-12) production by a subset of intratumoral DCs. Furthermore, triggering the T cell:DC crosstalk through non-canonical NF-κB agonism in combination with anti-PD-1 treatment has been found to perhaps potently enhance antitumor immunity. These data suggest that the responses to immunotherapy can be further improved through rational drug combinations that accentuate the crosstalk between lymphoid and myeloid. To understand the molecular determinants of immunotherapeutic response in glioblastoma (GBM), Zhao et al. used scRNA-seq to longitudinally profiled 66 patients, including 17 long-term responders, during standard therapy and after treatment with PD-1 inhibitors [30]. In their cohort, *PTEN* mutations are found to be significantly enriched in tumors from non-responders and might induce a distinct immunosuppressive TME. In particular, single-cell analysis reveals that this signature originates not from regulatory T (Treg) cells, but rather from tumor cells overexpressing *CD44*, a marker associated with cellular mobility and GBM aggressiveness. Another finding in their study is the distinct evolutionary patterns of responding and non-responding tumors under immunotherapy. Immunosuppression-associated gene sets are more active in non-responders before immunotherapy, but are more active in responders following treatment, supporting the role of tumor evolution in shaping the microenvironment. In the study of metastatic castration-resistant prostate cancer (CRPC), Jiao et al. established murine osseous and non-osseous (subcutaneous) CRPC models to identify the mechanisms underlying sub-optimal response to ICB [31]. By combining CyTOF, scRNA-seq, single-cell TCR sequencing, and multiplex cytokine profiling technologies, it is found that ICB fails to elicit an anti-tumor response in the bone CRPC model despite an increase in the number of intratumoral CD4 T cells, which are polarized to Th17 rather than Th1 lineage. Mechanistically, tumors in the bone have been observed to promote osteoclast-mediated bone resorption that releases transforming growth factor-β (TGF-β), which restrains Th1 lineage development. Through blocking TGF-β along with ICB, they have successfully increased the proportion of Th1 subsets, promoted clonal expansion of CD8^+^ T cells and subsequent regression of bone CRPC, as well as improved survival.

Recently, single-cell analysis has also been widely used in the identification of new potential checkpoint targets, especially for highly metastatic cancer types that are largely unresponsive to the existing checkpoint immunotherapies. For instance, Durante et al. used scRNA-seq to interrogate tumor and non-neoplastic single cells from 8 primary and 3 metastatic uveal melanoma (UM) samples [32]. They have found that tumor-infiltrating immune cells comprise a previously unrecognized diversity of cell types, including CD8^+^ T cells predominantly expressing the checkpoint marker *LAG3*, rather than *PDCD1* or *CTLA4*, suggesting that *LAG3* is a potential candidate for ICB in patients with high-risk UM. Their scRNA-seq V(D)J analysis also shows clonally expanded T cells and/or plasma cells in UM samples, indicating that tumor infiltrating immune cells are capable of mounting a response. These observations suggest that low tumor mutation is not the only explanation for the poor response of UM to checkpoint inhibitors. In another study aiming to garner an insight into tumor-specific immunomodulatory targets, Goswami et al. analyzed 94 patients representing five different cancer types, including those that respond relatively well to immune checkpoint therapy and those that do not, such as GBM, prostate cancer, and colorectal cancer [33]. Through CyTOF and scRNA-seq, a unique population of CD73^hi^ macrophages has been identified in GBM that persists after anti-PD-1 treatment. To further test if targeting CD73 would be important for a successful combination strategy in GBM, Goswami et al. also performed reverse translational studies using *CD73*^−/−^ mice. It is found that the absence of CD73 improves survival in a murine model of GBM treated with anti-CTLA-4 and anti-PD-1. Their data confirm CD73 as a specific immunotherapeutic target to improve antitumor immune responses to immune checkpoint therapy in GBM.

### Single-cell analysis of cellular therapies

**Figure 3:**
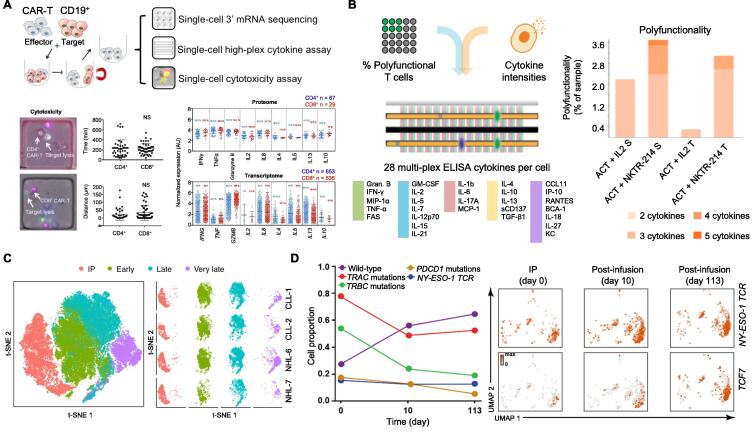
**Representative studies employing single-cell technologies to understand the functional characteristics of engineered cellular products in ACT** **A.** Using scRNA-seq, single-cell multiplex cytokine secretion assay, and live-cell imaging of cytotoxic activity to profile anti-CD19 CAR-T cell reactions upon antigen-specific stimulation. Single-cell data reveal that both CD4^+^ and CD8^+^ CAR-T cells are cytotoxic and a highly mixed Th1/Th2 cell response is observed [35]. **B.** Using single-cell IsoCode chip to analyze the persistence of adoptively transferred T cells with a kinetically engineered IL-2 receptor agonist. In both spleen (S) and tumor (T), cells treated *in vivo* with ACT + NKTR-214 have increased polyfunctionality [40]. **C.** Using TCR beta sequencing, integration site analysis, and scRNA-seq to demonstrate clonal kinetics and transcriptional profiling of CAR-T cells in patients. The t-SNE representation of 62,167 CD8^+^ CAR-T cells concatenated from the IP, early, late, and very late time points of four patients (CLL-1, CLL-2, NHL-6, and NHL-7) is generated using scRNA-seq data [41]. **D.** Using scRNA-seq to profile the first-in-human CRISPR/Cas9 edited TCR-T cells. The frequency of gene-edited T cells declines from IP, whereas the expression of genes associated with central memory (*TCF7*) increases over time [43]. Panel A is adapted from [35] with permission. Panel B is adapted from [40] with permission. Panel C is adapted from [41] with permission. Panel D is adapted from [43] with permission. ACT, adoptive cellular therapy; CAR-T, chimeric antigen receptor T; IsoCode, single cell antibody coated chip commercialized by Isoplexis; t-SNE, t-distributed stochastic neighbor embedding; IP, infusion product; CLL, chronic lymphocytic leukemia; NHL, non-Hodgkin lymphoma; TCR-T, TCR-engineered T.

Compared to studies on ICB, single-cell analysis has not been fully utilized in ACT studies. Several pioneering works have applied single-cell technologies to understand the functional characteristics of engineered cellular products. In 2017, Xue et al. employed a 16-plex cytokine microfluidic device to profile the secretome of CD19 CAR-T cells upon antigen-specific stimulation at the single-cell level [34]. Their results reveal that CAR-T cells exhibit a marked heterogeneity of cytokine secretion and polyfunctional (secreting more than two types of cytokines) subsets specific to anti-CAR bead stimulation, providing a new platform for capturing intrinsic characteristics of CAR-T cells for correlative analysis. In addition, our lab presents the first comprehensive portrait of single-cell level transcriptional and cytokine signatures of the third-generation anti-CD19 CAR-T cells upon antigen-specific stimulation [35] (Figure 3A). By combining high-throughput scRNA-seq, single-cell multiplex cytokine secretion assay, and live-cell imaging of cytotoxic activity, we reveal that CD4^+^ and CD8^+^ CAR-T cells are equally effective in direct killing of target tumor cells and that the activation states of these CAR-T cells are highly mixed with Th1, Th2, and Treg responses in the same single cells and largely independent of differentiation status. Furthermore, granulocyte–macrophage colony-stimulating factor (GM-CSF) is produced from the majority of cells regardless of the polarization state, which contrasts CAR-T to autologous T cells. This work provides new insights into the biology of CAR-T cell activation and a route to develop single-cell approaches for CAR-T infusion product quality assurance.

In a recent scRNA-seq evaluation of healthy donor-derived second-generation CAR-T cells, Boroughs et al. have identified that compared to CAR-T cells structured with CD28 co-stimulatory domain, CD19-targeted CAR-T cells bearing 4-1BB co-stimulatory domain have increased expression of human leukocyte antigen (HLA) class II gene, *ENPP2*, and IL-21 axis genes, but decreased expression of *PDCD1* [36]. They have also found an enrichment of central memory cell phenotype and fatty acid metabolism in CD8^+^ 4-1BB CAR-T cells. In another large-scale single-cell transcriptomic analysis of third-generation CAR-T cells that target both B cell maturation antigen (BCMA) and transmembrane activator, calcium modulator, and cyclophilin ligand interactor (TACI), Wang et al. have found that CAR products from three different donors exhibit a similar cellular composition, the manufacturing process of CAR-T cells induces effector-like transcriptional signatures, and the CAR-specific antigen exposure activates similar pathways as those triggered by TCR [37].

Single cell antibody coated chip commercialized by Isoplexis (IsoCode) has been broadly utilized to reveal the functional states of CAR-T products and their correlation with clinical response. In a study of patients with aggressive refractory non-Hodgkin lymphoma that were treated with CAR-T cells, Rossi et al. used IsoCode chip to find the association between pre-infusion CAR-T cell polyfunctionality and clinical outcomes [38]. To understand the factors that are associated with clinical response in anti-CD19 CAR-T-cell therapy, they have characterized product T cells using polyfunctionality strength index (PSI), based on the frequency and production levels of homeostatic/proliferative, inflammatory, chemotactic, regulatory, and immune effector molecules. The analysis shows that only 20%–25% of all product cells are polyfunctional upon stimulating CD19-expressing target cells, and polyfunctional profiles for both CD4^+^ and CD8^+^ T cells are composed of specific effector molecules, stimulatory/immune-modulating cytokines, and chemokines. The major finding of this work is that PSI, combined with conditioning-driven IL-15 or CAR-T cell expansion *in vivo*, is associated with post-CAR-T cell therapy outcomes. This exploratory study underscores the potential of using cell polyfunctionality as a key product attribute to evaluate the quality of pre-infusion CAR-T products, complementing other existing characteristics such as T-cell proliferative capability. In a study to investigate whether the functions of glypican 3-targeted CAR-T cells are affected by their antibody-binding properties, Li et al. created CAR-T cells based on the humanized YP7 (hYP7) and HN3 antibodies and used the 32-plex IsoCode to compare the polyfunctionality of these cells [39]. They have found that hYP7 CAR-T cells derived from both healthy donors and patients with hepatocellular carcinoma can stimulate robust activation and expansion of polyfunctional T cells, particularly through small subsets of CD8^+^ cytotoxic T cells that lyse tumor cells by inducing perforin/granzyme apoptosis pathway. These results verify that hYP7 CAR-T cells are more potent than HN3 CAR-T cells, which is consistent with the *in vivo* tumor elimination observations. In another work aiming to improve the proliferation, homing, and persistence of anti-tumor ACT cells, NKTR-214, a novel IL2Rβγ-biased cytokine, was designed to function as an alternative to conventional IL-2 [40] (Figure 3B). By comparing the intrinsic properties of T cells between ACT + NKTR-214 and ACT + IL-2 treated-mice, Parisi et al. have evaluated the polyfunctionality of the adoptively transferred cells using IsoCode. It is found that compared to ACT + IL-2, cells treated with ACT + NKTR-214 *in vivo* show increased polyfunctionality in spleen and tumor by 1.7-fold and 10-fold, respectively. These results support the ability of NKTR-214 to strongly activate a pool of cytotoxic effector T cells.

To depict the clonal kinetics and transcriptional programs that regulate the fate of CAR-T cells after infusion, Sheih et al. combined TCR beta sequencing, integration site analysis, and scRNA-seq to profile CD8^+^ CAR-T cells from infusion products and blood of patients [41] (Figure 3C). They find that clonal diversity of CAR-T cells is the highest in infusion products and declines following infusion, and clones that expand after infusion mainly originate from infused clusters with higher expression of cytotoxicity and proliferation genes. This work uncovers transcriptional programs associated with CAR-T therapy and demonstrates the potential for scRNA-seq to provide unique insights into the *in vivo* behavior of CAR-T cells after adoptive transfer.

Single-cell technologies have also been used in the characterization of TCR-T cell therapies. In a recent pilot trial designed to investigate the safety, feasibility, and antitumor efficacy of New York esophageal squamous cell carcinoma 1 (NY-ESO-1) targeted ACT with DC vaccination, with and without CTLA-4 blockade by ipilimumab, a CyTOF staining panel with 33 different surface and intracellular markers has been utilized to characterize the transgenic T-cell phenotypic subpopulations over time [42]. The results show that the infusion products in both cohorts contain large proportions of less differentiated central memory and effector memory phenotypes, while there is a shift to more terminally differentiated effector phenotypes in the post infusion recovery products. Very recently, the first-in-human phase I clinical trial using multiplex CRISPR-Cas9 editing to engineer T cells in three patients with refractory myeloma or sarcoma has been reported by Carl H. June and his team [43] (Figure 3D). In this work, scRNA-seq has been employed to characterize the transcriptomic phenotype of the genetically-engineered T cell products and their evolution over time in one patient. They compared the gene expression pattern between the infusion products and the post infusion product on day 10 and day 113. The results show a decline in the frequency of gene-edited T cells 10 days and 4 months after the infusion, and this decline occurs regardless of whether the cells have been transduced with the NY-ESO-1 TCR or not. However, the frequency of gene-edited cells is quite stable between day 10 and day 113.

## Identifying predictable biomarkers

A biomarker is a measurable biological indicator that can provide insights into a patient’s genetic makeup, the interactions between tumor and immune system, as well as the potential response to cancer immunotherapy, which can help doctors determine the most probable beneficial therapeutic approach for a particular patient. Biomarkers come in diverse categories. Pretreatment biomarkers can guide doctors’ decision-making regarding to diagnostic, prognostic, or predictive process, answering important questions about cancer type, expected outcome, potential response rate, and side effects. In contrast, biomarkers during and after treatment can provide short-term or extended monitoring that determines whether the treatment is working effectively or if the cancer is still in remission.

Several promising biomarkers have already been incorporated into clinical practice in current ICB. For example, tumor PD-L1 expression reflects an immune-active microenvironment and is the factor most closely correlated with response to anti-PD-1 blockade, due to its association with PD-1 and PD-L2 expression, [44]. PD-L1 expression detected by immunohistochemistry has become an FDA-approved companion diagnostic test for pembrolizumab treatment in non-small cell lung carcinoma (NSCLC), gastric/gastroesophageal junction adenocarcinoma, cervical cancer, and urothelial cancer [45], [46], [47], [48]. Tumor mutation burden (TMB) is another widely used biomarker to predict the efficacy of ICB across many cancer types [49], [50], [51]. High TMB represents higher levels of neoantigens, which are thought to incite more effective anti-tumor responses and can therefore help identify patients who can benefit from ICB. Additionally, the presence of TILs [52], immune gene expression signatures [53], and the diversity and composition of gut microbiome [54] have also been recognized as emerging predictive biomarkers.

These biomarkers are not without limitations. For example, there is a caveat that some patients with high PD-L1 expression may not respond well, while other patients who lack high PD-L1 expression may still respond. In the case of TMB, for several tumor types like Merkel-cell carcinoma and renal-cell carcinoma, patients with intermediate levels of TMB still exhibit a high response rate to ICB [55], [56]. By parsing out the role of cellular heterogeneity, profiling molecular networks and interactions, and establishing the whole‐cell models, single-cell technologies have the potential to deliver new insights into the identification and validation of disease-specific or therapy-specific biomarkers, enabling the prediction of response and implication of acquired immune resistance while monitoring immunotherapy-related toxicity. Table 2 lists the studies that employ single-cell technologies to identify biomarkers in immunotherapy.

**Table 2:** **Summary of the studies employing single-cell technologies to delineate the mechanisms of immunotherapy**

*Note*: TIM-3, T-cell immunoglobulin mucin 3; ADAR1, adenosine deaminase acting on RNA 1; BCR-seq, B cell receptor sequencing; FAS, Fas cell surface death receptor; EOMES, eomesodermin; MIP-1β, macrophage inflammatory protein 1 β; TLR3, toll-like receptor 3; MCPyV, Merkel cell polyomavirus.

### Predicting response to immunotherapy

Currently, the most promising outcome obtained in ICB is observed in the PD-1 treatment of melanoma and NSCLC patients, with an objective response rate of 40%–45% [57], [58], [59]. In clinical trials of CD19-targeted CAR-T adoptive therapy, although complete remission has been induced in 67%–93% of the patients with ALL, efforts to replicate such success to other malignancies have not achieved satisfactory outcomes yet, especially for solid tumors [60]. Considering the substantial number of immunotherapy-treated patients showing no beneficial response, the identification of reliable biomarkers that can inform clinical response will be critical to discriminate responders from non-responders before starting a costly and complex treatment, or providing potential non-responders with alternative therapeutic options.

**Figure 4:**
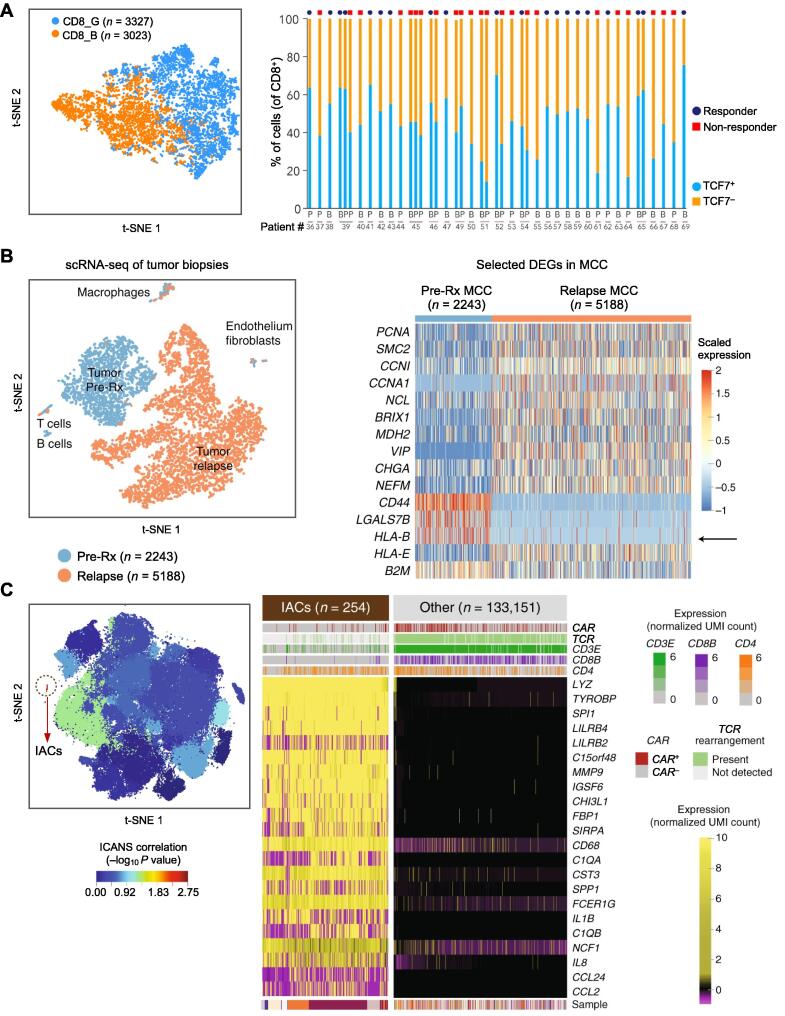
**Single-cell technologies have the potential to deliver new insights into the identification and validation of biomarkers during the immunotherapy treatment** **A.** scRNA-seq showing that *TCF7*^+^CD8^+^ T cell frequency in tumor tissue can predict response and better survival in melanoma patients treated with checkpoint inhibitor. Profiling of 6350 CD8^+^ T cells reveals two major cell states: CD8_G with increased expression of genes linked to memory, activation, and cell survival; CD8_B enriched for genes linked to cell exhaustion. More *TCF7*^+^CD8^+^ cells are found in responders, whereas *TCF7*^−^CD8^+^ cells are enriched in non-responders, indicating that the presence of *TCF7* could be a predictive factor of clinical response to the anti-PD-1 therapy [61]. B represents baseline; P represents post-therapy. **B.** scRNA-seq showing acquired cancer resistance to combination immunotherapy from transcriptional loss of class I HLA. t-SNE clustering indicates substantial transcriptional change from pre-treatment (Pre-Rx) to late relapse/acquired resistance (after 615 days). The heatmap of DEGs shows that MCC tumor cells have significantly downregulated *HLA-B* expression at acquired resistance [79]. **C.** scRNA-seq showing that cells with a monocyte-like transcriptional signature are associated with high-grade ICANS in patients treated with CAR-T. The significant cluster of IACs is circled. Genes that are most highly expressed in cells from the IAC cluster compared to cells from other clusters are shown in a heatmap [64]. Panel A is adapted from [61] with permission. Panel B is adapted from [79] with permission. Panel C is adapted from [64] with permission. HLA, human leukocyte antigen; MCC: Merkel cell carcinoma; ICANS, immune effector cell-associated neurotoxicity syndrome; IAC, ICANS-associated cell; UMI, unique molecular identifier.

To date, although not fully applied, several studies have described the pioneering use of single-cell technologies in biomarker discovery for response forecast of immunotherapy, and some T cell subpopulations harboring unique phenotype have been identified as potential predictive biomarkers. For instance, Sade-Feldman et al. profiled transcriptomes of 16,291 individual immune cells of 48 tumor samples from melanoma patients treated with checkpoint inhibitors [61] (Figure 4A). The presence of *TCF7*, a single transcription factor in CD8^+^ T cells, is found to be predictive of clinical response to ICB, suggesting that the state of T cells is critical for the induction of effective tumor immunity, rather than the number or their spatial distribution. Through screening by high dimensional clustering, Takeuchi et al. explored comprehensive immune cell responses associated with clinical benefits using peripheral blood mononuclear cells (PBMCs) collected from pre- and post-treatment samples from two different cohorts [62]. In the discovery set, it is found that a subset of central memory CD4^+^ T cells with CD27^+^FAS^−^CD45RA^−^CCR7^+^ phenotype is enriched in long-term survivors treated with anti-PD-1 monoclonal antibody, but not in non-responders. The same increase has also been observed in clinical responders in the validation set. In another study, Gide et al. performed transcriptomic and immune profiling on patients treated with anti-PD-1 monotherapy (*n* = 63) or combined anti-PD-1 and anti-CTLA-4 therapy (*n* = 57) [63]. It is identified that compared to non-responders, EOMES^+^CD69^+^CD45RO^+^ effector memory T cell phenotype is significantly more abundant in responders to combined immunotherapy. Very recently, Deng et al. have performed scRNA-seq of standard-of-care axicabtagene ciloleucel CAR-T infusion products administered to 24 patients with large B cell lymphomas [64]. They find that patients who have achieved a complete response at their 3-month follow-up have 3-fold higher frequencies of CD8^+^ T cells expressing memory signatures than patients with partial response or progressive disease.

While aforementioned studies have identified T cell compartments as predictive biomarkers and strategies to augment clinical response, other immune subsets may also contribute to anti-tumor immunity. For example, using CyTOF, Krieg et al. have identified that the frequency of CD14^+^CD16^−^HLA-DR^hi^ monocytes can be a potential predictor of progression-free and overall survival in response to anti-PD-1 immunotherapy [65]. Using the same approach, Subrahmanyam et al. have found that NK cells expressing both CD69 and MIP-1β likely play a critical role in the anti-tumor response triggered by anti-PD-1, whereas CD4^+^ and CD8^+^ memory T cell subsets are potential biomarker candidates for higher response rate to anti-CTLA-4 [66]. Similarly, Zemek et al. have compared gene expression patterns and cellular composition of mouse cancer models and found that responsive tumors have more infiltrating activated NK cells [67]. In addition, they have also identified that responsive tumors are characterized by an inflammatory gene expression signature, consistent with up-regulation of STAT1 and TLR3 signaling and down-regulation of IL-10 signaling. Recently, Helmink et al. have presented multi-omics data that support a role for B cells within tertiary lymphoid structures in the response to ICB in patients with metastatic melanoma and renal cell carcinoma [68]. It is found that B cell markers are the most differentially expressed genes in the tumors of responders versus non-responders. By assessing the potential functional contributions of B cells via bulk and single-cell RNA-seq analyses, Helmink et al. have demonstrated clonal expansion and unique functional states of B cells in responders. Moreover, CyTOF analysis has also shown that switched memory B cells are enriched in the tumors of responders.

Alterations in the DNA damage response or homologous recombination pathways could also influence response to ICB. Samstein et al. have recently reported differential effects of mutations in the homologous recombination genes *BRCA1* and *BRCA2* on response to ICB in mouse and human breast tumors [69]. Their data suggest that, compared to *BRCA1*, *BRCA2* deficiency is better associated with increased immunogenicity and improved response to ICB. scRNA-seq analysis further reveals that the superior therapeutic response observed in *Brca2*^null^ models and tumors relies on the cooperative actions of distinct T cell, natural killer, macrophage, and DC populations.

### Monitoring acquired immune resistance

In addition to challenges associated with the high unresponsive rate, the primary or acquired resistance that can preclude durable remissions stands as another barrier to immunotherapy cure. In the treatment with combination blockade of PD-1 and CTLA-4, patients with melanoma have the highest response rate compared to patients with other types of cancers. However, the response in 25% of melanoma patients is still not durable [59]. Similarly, in patients with NSCLC, delayed progression typically occurs within one year from the time of treatment [70]. In a longer follow-up of adult ALL patients treated with anti-CD19 CAR-T cells, approximately 30%–50% who achieve remission relapse within one year of treatment [71]. A complete understanding of the mechanisms underlying resistance is critical but complex, mainly due to a heterogeneous conglomerate of multiple cell types involved in tumor–immune interactions. For clinicopathologic factors associated with primary resistance to ICB, researchers have proposed and identified several now-characterized mechanisms, including lack of T-cell infiltration [52], elevated levels of baseline serum lactate dehydrogenase (LDH) [72], increased baseline tumor burden [73], insufficient neoantigens [49], low mutational burden [49], lack of PD-L1 expression [44], and other factors. For CAR-T cell therapy, failures of cell product manufacturing [74], limited *in vivo* CAR-T cell proliferation or persistence [75], and loss or modulation of the target antigens [76] are among the most commonly cited reasons for primary resistance to effective outcomes. In contrast, little is understood about the mechanisms underlying acquired resistance, which necessitates the use of novel single-cell strategies to identify biomarkers that have therapeutic implications for rescue.

Many studies have demonstrated the capability of single-cell technologies in identifying novel mechanism underlying immunotherapy escape. Oweida et al. have applied CyTOF and whole-genome sequencing to define changes in the TME of orthotopic murine head and neck squamous cell carcinoma (HNSCC) and identified mechanisms underlying resistance to radiotherapy (RT) and PD-L1 blockade [77]. They find that, after RT and PD-L1 treatment, the immune checkpoint receptor, TIM-3, is upregulated on CD8^+^ T cells and Tregs in tumors. They have also verified that treatment with anti-TIM-3 concurrently with anti-PD-L1 and RT leads to significant tumor growth delay, enhanced T-cell cytotoxicity, decreased proportion of Tregs, and improved survival in HNSCC. In another report, Ishizuka et al. have profiled CD45^+^ cells in the TME of melanoma tumor model using scRNA-seq analysis [78]. They have shown that loss of function of the RNA-editing enzyme adenosine deaminase acting on RNA 1 (ADAR1) in tumor cells profoundly sensitizes tumors to immunotherapy and overcomes resistance to PD-1 ICB caused by inactivation of antigen presentation. Additionally, the induction of sufficient inflammation in tumors that are sensitized to interferon can bypass the therapeutic requirement for CD8^+^ T cell recognition of cancer cells, which may provide a general strategy to overcome immunotherapy resistance. Recently, Paulson et al. have performed detailed investigations on two patients with Merkel cell carcinoma who had received the combination of T cell immunotherapy along with anti-CTLA4 ICB and developed late/acquired resistance [79] (Figure 4B). Profiling of the relapsed tumor using scRNA-seq reveals that when tumors relapse, there is a significant transcriptional downregulation of the HLA that restricts the targeted Merkel cell polyomavirus (MCPyV) epitope.

In a very recent investigation of mechanisms underlying CD19-negative relapse in one B cell ALL patient treated with CD19-targeted CAR-T therapy, Rabilloud et al. have used scRNA-seq to analyze leukemic cells before and after the therapy [80]. Their results demonstrate that CD19-negative leukemic cells are present before CAR-T cell therapy and thus the relapse results from the selection of these rare pre-existing CD19 negative subclones. Similarly, Da Vià et al. have also applied scRNA-seq to perform in-depth single-cell analysis of BCMA loss in a patient with multiple myeloma [81]. Consequently, they have identified selection of a clone with homozygous deletion of *TNFRSF17* (BCMA-coding gene) as the mechanism underlying immune escape.

One notable characteristic of acquired resistance is that it occurs on the level of individual cells, whereby tumor cells alter their gene expression in response to immune factors within the TME [82]. For example, tumor cells can upregulate PD-L1 expression in response to immune-related cytokines, such as IFN-γ released by T cells, hence limiting T-cell function [83]. Therefore, single-cell technologies can be further leveraged to profile T cell responses against mutant antigens with unprecedented resolution, enabling long-time follow up during the therapy to document patients’ natural immunological history and to find potential biomarkers.

### Managing immunotherapy-related toxicity

Immunotherapies can boost the natural defense against cancer in some patients, but they can also cause serious side effects even if less toxic than traditional chemotherapeutic agents. Checkpoint inhibition is associated with a unique spectrum of side effects that arise from general immunologic enhancement, including dermatologic, gastrointestinal, hepatic, endocrine, and other less common inflammatory events [84]. In CAR-T therapy, the associated toxicities are more acute and can be severe or even fatal. The most commonly observed toxicities are CRS, CAR-T cell related encephalopathy syndrome, and haemophagocytic lymphohistiocytosis or macrophage activation syndrome [85]. Notably, efforts are underway to establish general approaches for toxicity management, intensive monitoring, and accurate grading. For example, the American Society of Clinical Oncology (ASCO) has proposed both general guidelines and organ-system-specific recommendations for the management of adverse events associated with ICB [86], and the CRS grading scale has also been published as the American Society for Blood and Marrow Transplant consensus guidelines [87]. However, not all cases can be categorized in the clinic and, as such, intervention strategies need adjusting accordingly. For example, in CAR-T clinical trials designed to treat solid tumors, the on target/off tumor incident might cause more severe toxicity than the B cell aplasia broadly observed in the treatment of B cell malignancies, thus warranting novel approaches to mitigate adverse events [60]. Therefore, systematic investigations are necessary to define biomarkers to tell in advance which patients may be at greater risk for a specific immunotherapy.

Currently, several biomarkers have been identified as a way to assess the risk. In ipilimumab-treated patients with melanoma, higher on-treatment serum concentrations of IL-17 have been found in those who developed colitis symptoms [88]. In addition, increases in the expression of immunologically related genes can be observed to a greater degree in patients exhibiting gastrointestinal toxicity [89], and high lymphocyte and eosinophil counts present early in the course of therapy are thought to be potentially associated with a higher risk of immune-related adverse events [90]. In CAR-T therapy, the peak level of serum IFN-γ after CAR-T product infusion is positively correlated with the severity of CRS [91], and the higher peak level of serum IL-6 is associated with severe neurotoxicity [92]. Accordingly, early interventions have been developed to reduce the side effects, including the use of anti-IL-6 therapies and corticosteroids [93]. However, these data come from limited number of trials using bulk profiling methods, and therefore they may not be reliable enough to uncover novel and potentially unexpected biomarkers of severe toxicity.

Several research groups have applied single-cell platforms to evaluate the association between the expression level of biomarkers and the degree of adverse effects. In a study conducted by Das et al., circulating B cells before and after the first cycle of ICB therapy in 39 patients with advanced melanoma have been analyzed using scRNA-seq and BCR sequencing [94]. It is found that the combination checkpoint blockade therapy targeting CTLA-4 and PD-1 leads to a decline in the number of circulating B cells, and an increase in the number of CD21^low^ B cells and plasmablasts. They have also identified that the severity of an early decline in the number of B cells after therapy is directly correlated with the time to onset of immune-related adverse events as well as the grade of maximal toxicity, suggesting that preemptive strategies targeting B cells may reduce toxicities. In the correlation analysis between CAR-T characteristics and the therapy-associated side effects, Rossi et al., find that CRS with grade ≥ 3 is correlated with higher percentage of polyfunctional T cells, and both neurologic toxicity with grade ≥ 3 and antitumor efficacy are associated with increased number of polyfunctional IL-17A-producing T cells [38]. In another study, Deng and colleagues have identified a rare cell population with monocyte-like transcriptional features, and the higher fraction of this cell population is specifically associated with high-grade neurotoxicity syndrome [64] (Figure 4C).

## Conclusion and perspectives

The successful application of the immune checkpoint blockers and CD19-directed CAR-T cell therapy in treating multiple cancer types has revolutionized anticancer treatment and established immunotherapy as a potentially curative option for patients with advanced cancers. Recent years have seen a large number of single-cell technologies applied in characterizing systemic immune landscape, profiling intratumoral microenvironment, and identifying cancer cell heterogeneity, thus opening the doors to developing new generation of immunotherapy. In this review, we have discussed the spectrum of single-cell analytical techniques, including proteomic, transcriptomic, TCR/BCR sequencing, and multi-omics technologies, as well as their applications in furthering our understanding of immunotherapy in terms of underlying mechanisms and their association with therapeutic outcomes. We anticipate that these cutting-edge technologies will deliver new insights into the identification of cancer-specific biomarkers and prediction of clinical responses.

Despite the significant information provided by single-cell omics analyses, many challenges and limitations remain. As we see from the aforementioned reports, even when the same type of cancer is evaluated, the results are not always consistent with each other. This incongruency may be due to a combination of the heterogeneity of patients’ conditions and cancers, the complicated experimental design, and different computational methods used in the analyses. Another limitation present in the current state of immunotherapy biomarker discovery is the lack of systemic validations in larger patient cohorts, which is indispensable to translate the knowledge into the bedside companion diagnostics. To build on the potential biomarkers, we can design independent, blinded validation trials involving larger, multicenter cohorts of patients, and then test whether the rate of durable responses increases among selected patients.

Furthermore, one of the major weaknesses of conventional single-cell techniques is tissue dissociation during the cell processing, which leads to the loss of information about cell-to-cell interactions. As the field progresses, we envision technology-wise breakthrough not only to integrate multi-omics information from the same single cell, but also to dissect the crosstalk of heterotypic cellular complexes by sequencing physically-isolated cells. Several studies have been reported recently to achieve this. For example, an approach for sequencing physically interacting cells (PIC-seq) has been proposed to systematically map *in situ* cellular interactions and characterize their molecular crosstalk [95]. Using PIC-seq, T cell–DC interactions have been investigated *in vitro* and *in vivo* to profile interaction specificity and activated gene expression programs. Similarly, another novel method using a hierarchical loading microwell chip (HL-Chip) has also demonstrated its ability for aligning multiple cells of different types and/or microbeads at the single-cell resolution in a high-throughput process [96]. The interactions between *in vitro*-transduced NY-ESO-1-specific TCR-T cells and tumor cells have been evaluated and the results indicate that killing efficiency is a consequence of contact stability influenced by TCR ligand quality. These technologies have a great potential to increase our knowledge about the complex immune–tumor interaction networks during immunotherapies, thereby facilitating the discovery of new targets or design of combined therapy to enhance therapeutic benefits.

Much like pair-wise interactions and signaling, spatial location of individual cells in the functional tissue niches is also missing in traditional single-cell analyses. The field of spatial transcriptomics emerges to address this challenge either by fluorescence *in situ* hybridization (FISH)-based methods or NGS-based platforms. The first category, such as seqFISH [97], seqFISH+ [98], MERFISH [99], and STARmap [100], uses spectral barcoding and sequential imaging to localize nucleic acid targets, which is technically demanding and requires a lengthy repeated imaging process. Recently developed Slide-seq [101], HDST [102], and Visium commercialized by 10X Genomics use DNA barcoded beads to capture mRNAs from tissue and then perform unbiased, genome-scale molecular mapping through sequencing. These methods can provide high-spatial-resolution (down to 2 μm) and do not require sophisticated imaging system, although the number of detected genes is relatively low and the lateral diffusion of free mRNAs is unavoidable. In a recent effort to address these shortcomings, a fundamentally new technology, microfluidic Deterministic Barcoding in Tissue for spatial omics sequencing (DBiT-seq), has been developed to provide genome-scale information at a very high resolution [103]. As a highly versatile tool, DBiT-seq can combine different reagents for multiple omics measurements and can directly work on an existing fixed tissue slide.

However, most of the current whole-genome level spatial-omics platforms do not strictly resolve single cells and can only deal with fresh frozen tissue sections. To take single-cell spatial sequencing to the immuno-oncology bedside, major progress in technique harmonization will be indispensable. For example, the single-cell spatial methodology that is capable of obtaining genome-scale information from biobanked formalin-fixed, paraffin-embedded (FFPE) tissue samples would offer an invaluable resource for clinical research. In the foreseeable future, we envision that, through the integration with scRNA-seq data, spatial-omics analysis will broadly penetrate the field of immuno-oncology to dissect the TME heterogeneity and tumor–immune interactions, bringing about more rigorous identification of predictive biomarkers for immunotherapy response.

Ultimately, the goal of discovering biomarkers for immunotherapy applications at the single-cell level already has a solid foundation and encouraging prospects, some of which have been translated into benefits for patients. Further studies are needed to validate these biomarkers, and systemic improvement and optimization at the single-cell level are anticipated to enable biomarker discovery with higher precision, to aid in monitoring of disease-specific or patient-specific side effects, and to support the development of prophylactic strategies.

## CRediT author statement

**Zhiliang Bai:** Investigation, Resources, Writing - original draft, Writing - review & editing. **Graham Su:** Investigation, Resources, Writing - review & editing. **Rong Fan:** Conceptualization, Supervision, Writing - review & editing. All authors read and approved the final manuscript.

## Competing interests

Rong Fan is a co-founder of IsoPlexis, Singleron Biotechnologies, and AtlasXomics, as well as a member of their scientific advisory boards with financial interests, which could affect or have the perception of affecting the author’s objectivity. The interests of Rong Fan have been reviewed and managed by Yale University Provost’s Office in accordance with the University’s conflict of interest policies.

## Supplementary material

Supplementary File S1 **General introduction of the most widely used single-cell technologies for proteomic, transcriptomic, TCR/BCR, and multi-omics profiling**
