# Supplementary material for: Single-cell Analysis Technologies for Immuno-oncology Research: from Mechanistic Delineation to Biomarker Discovery
Source: Genomics Proteomics Bioinformatics. 2021 May 14;19(2):191–207. doi: 10.1016/j.gpb.2021.02.004 (PMC8602396; doi:10.1016/j.gpb.2021.02.004)
Supplement: Supplementary File S1 — General introduction of the most widely used single-cell technologies for proteomic, transcriptomic, TCR/BCR, and multi-omics profiling [file mmc1.docx]

**File S1 General introduction of the most widely used single-cell technologies for proteomic, transcriptomic, TCR/BCR, and multi-omics profiling**

A large number of single-cell technologies have leveraged advances in dissecting intratumoral and intertumoral heterogeneity, assessing individual recurrent neoantigens, and predicting patients’ responses to specific immunotherapy. Many of these technologies have been optimized to profile the tumor microenvironment and the immune system from the perspective of the genome, epigenome, transcriptome, proteome, and metabolome. In this supplementary section, we focus primarily on the technologies geared toward studying proteomics, transcriptomics, and multi-omics, which are the most common and effective methods for identifying heterogeneity in cancer patient samples.

**Proteomic analysis methods**

Proteins represent the main functional machinery of cells. The expressed proteomes varying from cell to cell execute a repertoire of important cellular functions including DNA replication, molecular transportation, cellular differentiation, signal transduction, and metabolic reactions [1,2]. Specifically, through the secretion of effector or signaling proteins, a myriad of immune functions, from targeted killing and self-renewal to the recruitment of other immune cell types and promotion or inhibition of local inflammation are executed to induce antitumoral response [3]. To provide more comprehensive characterization of the immune and cancer-associated cells, single-cell technologies aimed at profiling proteomics have been widely used to infer biological mechanisms, discover new biomarkers, and monitor cell-to-cell communications in clinical immunotherapies.

Traditionally, flow cytometry is one of the most versatile tools for studying single cells in immunology. Invented in the 1970s, this fluorescence-based assay can simultaneously measure up to 17 proteins from individual cells suspended in a solution [4]. Using instrumentation that directs a single stream of cells past a laser source and detects the resulting scatter and emission of light energy, a high-throughput rate of up to 10,000 cells per second can be achieved. Starting here, the number of target cells, the amount of a specific protein, and the functional activity can be obtained and analyzed to provide insights into the identification of cellular phenotypes. Equipped with a cell sorter, this technology can also be used to physically sort cells into separate containers based on the predefined fluorescent trigger. This process is called fluorescence activated cell sorting (FACS) [5]. From preclinical to early phase clinical trials, there is a growing interest in collecting more information for better prediction of the candidate drugs and identification of risks before an expensive, late-stage clinical trial, in which flow cytometry can be an indispensably powerful platform to monitor immune functions and immunotoxicity [6]. In fact, it is arguably the most widely-used single-cell analysis technology available at present and multiple clinical centers have established centralized flow cytometry facilities [7]. However, during the cell flowing, they are exposed to hydrodynamic pressure, laser beams, electrostatic charges, high voltage fields, and collisions with container surfaces, thereby influencing cellular recovery and integrity [8].

By conjugating antibodies with rare-earth mass tags to overcome the overlaps in fluorophore spectrum, mass cytometry (CyTOF) can further extend the profiling capacity to an upward of 100 different protein targets at the single-cell level [1]. As one of the core functional components, the elemental mass spectrometry can discriminate isotopes of different atomic weights with high accuracy, enabling significantly more cellular features to be detected simultaneously [9]. In 2011, this technology was first applied in a study of the immune system, in which 34 parameters in each individual cell in human bone marrow samples were obtained to provide an in-depth analysis of normal human hematopoietic and immunological signaling network [10]. Since then, CyTOF has been broadly employed to observe, quantify, and interrogate immune cell identity and behavior at the level of proteins, including identifying greater complexity in the CD8^+^ T cell compartment [11], performing high-dimensional phenotypic analysis of human T cells [12], and investigating B cell development in the healthy human bone marrow [13]. Currently, due to the dynamics of ion flight in the mass spectrometer, CyTOF can deal with cells at a throughput of 1000 cells/second, which lags behind that of fluorescence-based instruments. Additionally, it is still difficult to maintain small molecules and a binding agent associated with the cell, meaning that the measurement of molecular features with low expression levels is infeasible using CyTOF [14]. Moreover, similar to flow cytometry, it remains a challenge to recover living cells after analysis because cells are atomized and ionized.

Based on the immuno-enzyme technology originally developed for the enumeration of antibody-secreting cells [15,16], the ELISpot assay is a widely used, sensitive method that measures cytokine production at the single-cell level. The technique briefly includes the following steps [17]. Cells are cultured in membrane-bottomed wells pre-coated with cytokine-specific antibodies. Upon cell stimulation, locally-released cytokines will be captured by antibodies and accumulate in their surrounding environment. After applying a secondary antibody and fluorophore labels or utilizing an enzymatic reaction, trapped cytokine molecules can be detected. Finally, sharp blue/purple spots, each related to a cytokine-secreting individual cell, are visualized and counted on an inverted microscope or a reading system. By utilizing ELISpot, the frequency of cytokine-producing cells can be determined, which allows for the recording of these frequencies during different stages of anti-cancer treatments. In measuring peptide-specific T-cell responses in immunotherapy trials, this technique has been the gold standard for many years [18], with interferon-γ (IFN-γ) being the most commonly assayed cytokine [19].

The aforementioned techniques are still limited in throughput and sensitivity, and can only measure a relatively small number of proteins in single cells [20,21]. Microfluidic platforms have revolutionized proteomic analysis by providing highly sensitive and multiplexed single-cell assay, which has been comprehensively reviewed by Deng and his colleagues [22]. Here, we will only introduce several representative studies. One of them is the single-cell barcode chip (SCBC) developed by Ma et al. in 2011 [23]. Using control valves, single cells are isolated into thousands of nanoliter-volume microchambers. The secreted proteins are captured by a spatially DNA-encoded antibody library (DEAL) barcode array designed to fit a full panel of antibodies in each microchamber. Using this device, they have successfully measured 13 different effector molecules secreted by tumor antigen-specific cytotoxic T lymphocytes. In 2015, Lu et al. further optimized and simplified this device by directly isolating single cells into microchambers via gravity [24]. By combining spectral encoding with 3 different fluorescent colors and spatial encoding with 15 stripes, they achieve a codetection of 42 immune effector proteins secreted from single cells, representing the highest multiplexing to date for a single-cell protein secretion assay. Furthermore, this device has been applied to the study of functional heterogeneity in cell signaling, immune responses, tumorigenesis, and CAR-T cellular therapies [25–28].

In addition to the conventional microvalve, microwell, or droplet-based methodologies, Kennedy et al. recently developed a nanopore-driven technology to read the secretome from single cells [29]. When single molecules translocate through a nanopore in a thin inorganic membrane, electrolytic current blockades will be induced and can be measured to dynamically monitor and analyze secretions from single cells. In their pilot study, it is demonstrated that the distribution of blockades can be used to differentiate three different cancer cell lines (U937, MDA-MB-231, and MCF-7) in real time. Importantly, the distinctive blockades associated with the chemokine CCL5, a prognostic factor for disease progression in breast cancer, along with other low-mass biomarkers of breast cancer (PI3, TIMP1, and MMP1), have been identified.

Although the protein profiling methods have led to the discovery and characterization of major and minor cell types in the mammalian immune system [30], prior knowledge or guesswork, such as surface proteins, is required for protein-specific antibodies design, thereby limiting their applications to a detection of few parameters.

**Single-cell transcriptome profiling technologies**

In the studying of immunology, physiology, or pathology, the profiling of complete range of mRNA transcripts at the single-cell level can provide us valuable information to comprehensively dissect multicellular tissues, characterize cell to cell heterogeneity, and identify novel cell types in health and disease samples [31]. Technically, the major challenge of single-cell transcriptome profiling is the extremely low abundance of RNA, which is approximately 10 pg in a typical mammalian cell [32]. In the last two decades, a large number of breakthroughs in cell capture, reverse transcription efficiency, and cDNA amplification have expanded our reach to analyze transcriptome in a high throughput and low-cost fashion.

Traditional commonly-used techniques for mRNA capture and transcriptome analysis include fluorescence in situ hybridization (FISH) and quantitative PCR (qPCR). FISH allows for the observation of genetic alterations, such as rearrangements and translocations, through the localization of mutant genes or their respective mRNAs within individual cells, which is highly useful for identifying crucial markers in certain cancers [33,34]. qPCR offers a fluorescent quantification of single-cell gene expression without the need of complicated sample library preparation and has been proven as a promising method to unravel the heterogeneity of the developing immune system [35–37]. However, the implementations of both platforms require a prior knowledge of selected target genes, which limits the detection to only a finite number of genes and impedes the discovery of new unknown genes [38]. Therefore, there is a substantial interest to transition transcriptome profiling paradigm into unbiased single-cell techniques based on NGS.

In recent years, the advancement of single-cell RNA sequencing (scRNA-seq) has revolutionized our ability to analyze cell-specific transcriptome, which provides a holistic picture of diverse cell states and subsets as well as circuits within cellular populations and tissues [39]. Now, there are already many established scRNA-seq methods and platforms that can satisfy different requirements in the application of cancer immunobiology research, and it is possible to select the most suitable protocol depending on the sample size, the number of cells to be sequenced, and whether transcript counting or full-length mRNA sequencing is desired [40]. Here, we will summarize three mainstream scRNA-seq methods based on how single cells are captured or isolated, and categorize them into plate-based, droplet-based, and microwell-based groups.

Some early developed scRNA-seq platforms, such as SMART-seq [41], SMART-seq2 [42] and STRT-seq [43] normally employ micropipettes or FACS to isolate individual cells into 96-well plates, which can analyze 50–500 singles in each run. Although these micromanipulation approaches allow for a flexible experimental schedule and can profile any kinds of cell independent of type or size, the reverse transcription step needs to be carried out on individual microwells leading to higher molecular reagent cost, increased time-consumption, and low throughput. With workflow optimization and automation, in 2012, Fluidigm introduced the world’s first automated solution for single-cell genomics research, called C1. This innovative C1 system carefully isolates single cells into individual reaction chambers in the exclusive Fluidigm integrated fluidic circuit (IFC). The optically clear IFC can automatically stain captured cells and examine them by microscopy for viability, surface markers, or reporter genes, followed by cell lysis and template preparation. Currently, up to 800 cells can be analyzed at a time with high quality gene readouts, although it does have a relatively high cost per cell. To further decrease the reagent and labor cost while increase the scale of each experiment, Hashimshony et al. proposed CEL-Seq, a method for overcoming the limitation by barcoding and pooling samples before linearly amplifying mRNA with the use of a single round of *in vitro* transcription, providing both multiplexing and reproducibility [44]. By combining this barcoding strategy with FACS sorting, a handful of plate-based methods, including MARS-seq [45] and CEL-seq2 [46], can significantly reduce the hands-on time and cost without the compromise of lower sensitivity and precision.

The previously mentioned methods have allowed for the analysis of at most several thousand single-cells. However, in order to have enough resolution to resolve cell heterogeneity in the tumor as well as to identify lower represented cell populations like cancer initiating cells, the capacity of extensive sampling of cells is indispensable to fully understand the complexity of tumor microenvironment. In 2015, the advent of Drop-seq [47] and InDrop [48] platforms stood up to meet the needs. Both of them use microfluidic devices to encapsulate single cells, correlative reagents, and either barcoded mRNA capture beads or barcoded primers coated hydrogel microspheres into oil droplet. The oligonucleotide primers are directly synthesized onto beads, which contain (1) a constant sequence used as a priming site for downstream PCR and sequencing; (2) a ‘‘cell barcode’’ to digitally count the number of mRNA transcripts of each gene ascertained in each cell; (3) a Unique Molecular Identifier (UMI) to identify PCR duplicates and (4) an oligo-dT sequence for capturing polyadenylated mRNAs. Thanks to this key unique feature, the extracted barcoded material can be pooled in a single molecular reaction, facilitating the downstream library preparation. After cell lysis, the beads are retrieved and pooled for reverse transcription (RT) in Drop-seq, while this step is performed within the drops for InDrop. Currently, the droplet-based methods have become the most popular choice in the application of mapping large-scale single-cell atlases [49–51], mainly because it enables highly parallel transcriptomics profiling of tens of thousands single cells while still being economical. However, the increased breadth of this platform also makes compromises with reduced sensitivity. Compared with other existing methods, droplet-based approaches typically have lower transcript recovery (3%–10% *vs*. 10%–20%) [52].

Although the incessant workflow of Drop-seq allows for efficient single cell isolation, it also induces low cell-bead pairing efficiency that impedes its adoption for limited amount of patient samples. Further, the requirement of peripheral equipment limits their portability for remote clinical centers. Alternatively, microwell-based devices provide the scRNA-seq community a simplified strategy that is costless, portable, suitable for rare cell populations, and compatible with cell culture and imaging. One of the representative microwell-based platforms is Seq-well [53]. This device confines single cells and barcoded poly(dT) mRNA capture beads in a PDMS array of ~86,000 subnanoliter wells. By specifically adjusting the microwell dimensions, over 95% single-beads loading efficiency can be achieved. Another key advantage of Seq-Well is the use of selective chemical functionalization to facilitate reversible attachment of a semipermeable polycarbonate membrane in physiologic buffers, which enables rapid solution exchange, improve transcript capture, and reduce cross-contamination. The sequencing of human mouse species mixture and human peripheral blood mononuclear cells (PBMCs) have verified that this method can provide comparable performance with Drop-seq or 10x Genomics in terms of transcript capture efficiency, single-cell resolution, and detection sensitivity. Recently, we have developed a microwell-based platform called scFTD-seq [54] that uses freeze and thaw to lyse cells. Besides the satisfactory technical performance compared with other state-of-the-art methods, scFTD-seq offers format flexibility with a simplified, widely adoptable workflow that allows sampling at the distributed sites and downstream processing at centralized facilities, which enables the wide-spread adoption beyond academic laboratories.

Despite improvements of experimental scale, sensitivity, data quality, and single cell isolation methods, most of the above-mentioned protocols only sequence the 3’ end and fail to obtain the information of transcript variants. It has been widely demonstrated that alternative splicing plays an important role in tumor progression and immune surveillance, thereby having a great impact on immunotherapy responsiveness [55–58]. For example, it is found that in the treatment with B-cell acute lymphoblastic leukemias, the convergence of acquired mutations and alternative splicing of CD19 induces resistance to the CD19 targeted CAR-T immunotherapy [59]. So, it is highly anticipated that the full transcript sequencing can be integrated into current high-throughput and multiplexed workflows to provide a complete understanding of tumor biology at the single-cell level.

**Single-cell TCR and BCR sequencing**

Human T cells and B cells play a critical role in the adaptive anti-tumor immune responses by exert their abilities to recognize an infinite range of self or foreign antigens [60]. This functional flexibility is dependent on the diverse expression of B cell receptors (BCR) or T cell receptors (TCR), which governs the development, survival, and activation of these lymphocytes. In general, the repertoires of TCR or BCR change during the pathogenesis of oncological diseases, so the sequencing of TCR or BCR can provide high-resolution insights into the adaptive immune response, and thereby facilitating the development of diagnostic and monitoring tools, as well as the effectiveness of immunotherapies. However, the conventional bulk methods cannot elucidate the native TCRα:TCRβ or IgH:IgL pairs in individual cells, which really reflect their biological function *in vivo* and can only be achieved with single-cell analysis.

One of the early attempts enabling the integration of TCR specificity with single T cell function is performed by Han and his colleagues [61]. By using a nested PCR approach followed by deep sequencing, they have devised a strategy for the simultaneous sequencing of rearranged TCR genes and multiple functional genes in single T cells. Although the approach allows for highly efficient TCR determination and multiparametric phenotypic analysis, the use of 96-well PCR plates to sort cells limits its throughput. Another PCR-based methodology is developed by Turchaninova and his colleagues. They have reported a new RT-PCR suppression technique that enables the selective fusion of the native pairs of amplified TCR α and β chain genes for complex samples [62]. Thousands of single cells can be generated within oil-in-water emulsion droplets, which drastically increases the number of cells being processed in parallel. Using a similar emulsion droplets approach, Dekosky et al. have developed a workflow that permits sequence analysis of paired heavy- and light-chain from over 2 million B cells with demonstrated pairing precision of >97%, which enables paired antibody repertoire profiling at great depth in large study cohorts [63]. Additionally, Busse et al. have also devised a high throughput platform that employs a two-dimensional barcoded primer matrix to combine IgH and IgK/IgL chain gene single-cell PCR with next generation sequencing, allowing for the parallel repertoire analysis of over 46,000 individual B cells [64].

The development of scRNA-seq technologies opens a new perspective for TCR or BCR analysis. Stubbington et al. have developed a novel computational method known as TraCeR to accurately link T cell specificity with functional response by revealing clonal relationships between cells alongside their transcriptional profiles through the full-length scRNA-seq of individual T lymphocytes [65]. Their approach provides sequences in good concordance with those generated by the PCR-based method and is able to successfully reconstruct TCR sequences from a variety of sequencing depths and read types. Similarly, Redmond et al. have described scTCRseq to computationally identify single cell’s V(D)J gene rearrangements and recover the entire receptor sequence including constant region and nucleotides inserted and deleted at junctions [66]. However, a common approach that can provide full-length sequencing is plate-based Smart-Seq2 [42], which has its intrinsic drawbacks of low throughput, high cost, and time consuming, as we summarized in previous section. Alternatively, Afik et al. have developed ‘TCR Reconstruction Algorithm for Paired-End Single cell’ (TRAPeS), a software capable of accurately reconstructing TCRs from short-read paired-end sequencing libraries of single cells [67]. In the characterization of CD8+ T cell response in humans and mice, they have demonstrated that the algorithm is accurate and more sensitive than existing approaches although the throughput of this method is still quite limited as it can only investigate several hundreds of single cells. Therefore, it is anticipated that massively parallel and multiplexed assays will be available to enable paired transcriptomic and immune repertoire profiling.

Very recently, Singh et al. have described a novel method, termed Repertoire and Gene Expression by Sequencing (RAGE-Seq), to accurately pair gene-expression profiles from thousands of single cells with targeted full-length mRNA sequences combining high-throughput droplet-based scRNA-Seq workflows with Oxford Nanopore sequencing [68]. During the library preparation, they split some full-length single-cell 3’-tag cDNA prior to fragmentation and selectively enriched BCR and TCR cDNA transcripts using targeted hybridization capture. Then, the enriched antigen-receptor molecules are sequenced by long-read Oxford Nanopore to obtain both the 3’ cell-barcode and the 5’ V(D)J sequence. In parallel, the remaining cDNA is fragmented and sequenced using short-read Illumina sequencing to obtain the gene expression. Finally, the two cell barcodes from long-read sequencing and short-read sequencing are matched, and the transcriptome profiles for each individual cell can be linked with full-length antigen–receptor sequences. They have demonstrated the power of RAGE-seq by accurately tracking the clonal evolution of 7138 single cells sampled from the primary tumor and draining lymph node of a breast cancer patient, which is applicable to the study of cancer and could have significant translational applications.

**Single-cell multi-omics technologies**

A cell’s functional state is determined collectively by the interplay between its genome, transcriptome, proteome, and epigenome. Through the combination of single-cell assays to measure multiple types of molecules derived from an individual cell, as opposed to merging various mono-omics data obtained from different single cells, multi-omics technologies can provide a more complete set of information of each cell and deepen our understanding of the complex mechanisms governing cancer remission or progression. Furthermore, measuring the associations between multiple omics data sets allows for the identification of underlying cellular heterogeneity and enabling more profound biological insights in the era of precision medicine. Building on the development of microfluidics and cell barcoding techniques, many approaches have emerged to capture multi-channel information from the same cell, including the integrated study of epigenomes and transcriptomes, genomes and transcriptomes, as well as transcriptomes combined with targeted proteomes. Here, we will briefly review the recently developed multi-omics techniques, and readers can turn to other papers for more comprehensive overviews [39,69–71].

The genomic variation between cells can be linked unambiguously to the variations in their transcript levels through single-cell methods that can simultaneously capture DNA and RNA. The first integrated platform permitting the co-measurement of whole genome amplification and whole transcriptome amplification is reported by Han and his colleagues [72]. They have developed a microfluidic-facilitated approach that allows for the controlled separation of cytoplasmic and nuclear contents of a single cell followed by on-chip amplification of genomic DNA and cytoplasmic mRNA. However, the loss of RNA or DNA due to non-specific binding to PDMS channel walls is still unavoidable. Later, Dey et al. have devised DR-seq [73], a method that does not involve physical separation of the nucleic acids before amplification, thereby minimizing losses and chances of contamination. After DNA and RNA are released, the sample is split into two halves and processed separately to amplify and sequence gDNA or cDNA. Another widely used protocol is G&T-seq [74], in which a single cell's poly-A tailed RNA is separated from its genomic DNA using a biotinylated oligo-dT coated magnetic beads and both the genome and the transcriptome are then amplified in parallel and sequenced. Recently, Han et al. have reported a novel method for the simultaneous isolation of genomic DNA and total RNA (SIDR) from single cells [75]. They have utilized a hypotonic lysis approach to preserve nuclear lamina integrity and subsequently capture the cell lysate using antibody-conjugated magnetic microbeads, which enables reliable and efficient separation of genomic DNA and total RNA from single cells.

Genetically identical tumor cells may have different DNA methylation during complex biological processes, and so techniques that profile a cell’s epigenome and transcriptome can reveal the correlation of methylation differences with gene transcription variance. Based on the G&T-seq procedure to isolate DNA and RNA, scM&T-seq is the first reported method that combines DNA methylome and transcriptome profiling into one protocol [76]. Here, single cell reduced representative bisulfite sequencing (scRRBS [77]) is applied to the purified genomic DNA to generate methylomes and transcriptomes from the same single cells. For scMT-seq [78] and scTrio-seq [79], the cell membrane is gently lysed to keep the nucleus intact, and the cytoplasmic mRNA is separated from genomic DNA. In both technologies, the nuclear genome is processed using scRRBS, while the mRNA library is prepared using modified Smart-seq2 in scMT-seq and Tang’s protocol [80] in scTrio-seq. Above mentioned methods all capture the methylation as epigenome information, and recently, Clark et al. have developed scNMT-seq for parallel chromatin accessibility, DNA methylation, and transcriptome profiling, providing a broader view by allowing multiple layers of the epigenome to be read [81]. scNMT-seq uses a GpC methyltransferase to label open chromatin followed by bisulfite and RNA sequencing. By applying it to differentiating mouse embryonic stem cells, they have found links between all three molecular layers and have revealed dynamic coupling between epigenomic layers during differentiation. Most recently, Liu et al. have reported scCAT-seq, a technique for simultaneously assaying chromatin accessibility and the transcriptome within the same single cell [82]. Using this platform, they have generated the first integrated single-cell epigenomic and transcriptomic maps during pre-implantation embryo development.

Understanding the interaction between transcripts and proteins on a genome-wide scale is important in comprehending post-transcriptional processes and revealing functional phenotypic cell states that remain undercover from scRNA-seq techniques. Darmanis et al. uses FACS to isolate single cells, followed by lysing and dividing cells for separate RNA or protein analysis. Proteins are probed using a homogeneous affinity-based proximity extension assay and RNAs are reverse transcribed, followed by microfluidic qPCR reading [83]. Another technology leverages the DNA polymerase activity of reverse transcriptase to simultaneously perform proximity extension assays and complementary DNA synthesis in the same reaction, enabling parallel characterization of 38 proteins and 96 transcripts [84]. However, the applications of both methods are limited due to their low throughput. In PLAYR (proximity ligation assay for RNA), pairs of DNA oligonucleotide probes are designed to hybridize to two adjacent regions of target transcripts, and proteins are labeled with antibodies conjugated to metal isotopes. Making use of mass cytometry, this approach enables the simultaneous quantitative acquisition of more than 40 different proteins and RNAs routinely in thousands of cells per second [85]. In CITE-seq and REAP-seq, oligonucleotide-conjugated antibodies are used to integrate cellular protein and transcriptome measurements into an efficient, single-cell readout, which enables simultaneous quantification of about 100 proteins along with tens of thousands of RNA transcripts [86,87]. After reverse transcription and amplification, the barcoded cDNA and antibody libraries can be separated by size, so the protein quantification has almost no detriment to the quality of the RNA. Notably, both methods are readily adaptable to all the droplet-, microwell-, and combinatorial-indexing-based high-throughput single-cell sequencing technologies and are also fully compatible with a commercialized 10x platform. Although the techniques are now limited to cell surface proteins detection, theoretically, they can also be extended to the profiling of cytosolic and even nuclear proteins by applying a permeabilization before antibody incubation and staining.

**References**

[1] Doerr, A. Single-cell proteomics. Nat Methods 2019;16:20.

[2] Kim MS, Pinto SM, Getnet D, Nirujogi RS, Manda SS, Chaerkady R, et al. A draft map of the human proteome. Nature 2014;509:575–81 .

[3] Ma C, Fan R, Elitas M. Single cell functional proteomics for assessing immune response in cancer therapy: technology, methods, and applications. Front Oncol 2013;3:133.

[4] Perfetto SP, Chattopadhyay PK, Roederer M. Seventeen-colour flow cytometry: unravelling the immune system. Nat Rev Immunol 2004;4:648–55 .

[5] Cossarizza A, Chang HD, Radbruch A, Akdis M, Andrä I, Annunziato F, et al. Guidelines for the use of flow cytometry and cell sorting in immunological studies. Eur J Immunol 2017;47:1584–797.

[6] Davis MM. A prescription for human immunology. Immunity 2008;29:835–8.

[7] Maecker HT, McCoy JP Jr, FOCIS Human Immunophenotyping Consortium, Amos M, Elliott J, Gaigalas A, et al. A model for harmonizing flow cytometry in clinical trials. Nat Immunol 2010;11:975–8.

[8] Marie D, Le Gall F, Edern R, Gourvil P, Vaulot D. Improvement of phytoplankton culture isolation using single cell sorting by flow cytometry. J Phycol 2017;53:271–82.

[9] Bjornson ZB, Nolan GP, Fantl WJ. Single-cell mass cytometry for analysis of immune system functional states. Curr Opin Immunol 2013;25:484–94.

[10] Bendall SC, Simonds EF, Qiu P, Amir ED, Krutzik PO, Finck R, et al. Single-cell mass cytometry of differential immune and drug responses across a human hematopoietic continuum. Science 2011;332:687–96.

[11] Newell EW, Sigal N, Bendall SC, Nolan GP, Davis MM. Cytometry by time-of-flight shows combinatorial cytokine expression and virus-specific cell niches within a continuum of CD8+ T cell phenotypes. Immunity 2012;36:142–52.

[12] Newell EW, Sigal N, Nair N, Kidd BA, Greenberg HB, Davis MM . Combinatorial tetramer staining and mass cytometry analysis facilitate T-cell epitope mapping and characterization. Nat Biotechnol 2013;31:623–9.

[13] Bendall SC, Davis KL, Amir ED, Tadmor MD, Simonds EF, Chen TJ, et al. Single-cell trajectory detection uncovers progression and regulatory coordination in human B cell development. Cell 2014;157:714–25.

[14] Spitzer MH, Nolan GP. Mass cytometry: single cells, many features. Cell 2016;165:780–91 .

[15] Czerkinsky CC, Nilsson LA, Nygren H, Ouchterlony O, Tarkowski A. A solid-phase enzyme-linked immunospot (ELISPOT) assay for enumeration of specific antibody-secreting cells. J Immunol Methods 1983;65:109–21.

[16] Czerkinsky C, Andersson G, Ekre HP, Nilsson LA, Klareskog L, Ouchterlony O. Reverse ELISPOT assay for clonal analysis of cytokine production I. Enumeration of gamma-interferon-secreting cells. J Immunol Methods 1988;110:29–36.

[17] Yssel H, Wijdenes J, Malefyt RdW, Mathieu JF, Pène J. Measuring human cytokine responses. Methods Microbiol 2010;37:439–96.

[18] Whiteside TL, Zhao Y, Tsukishiro T, Elder EM, Gooding W, Baar J. Enzyme-linked immunospot, cytokine flow cytometry, and tetramers in the detection of T-cell responses to a dendritic cell-based multipeptide vaccine in patients with melanoma. Clin Cancer Res 2003;9:641–9.

[19] Ansari MJ, Strom TB. Novel diagnostics in transplantation. In: Himmelfarb J, Sayegh MH, editors. Chronic kidney disease, dialysis, and transplantation. 3rd ed. Philadelphia: W.B. Saunders; 2010, p.609–19.

[20] Spiller DG, Wood CD, Rand DA, White MRH. Measurement of single-cell dynamics. Nature 2010;465:736–45.

[21] Taylor RJ, Falconnet D, Niemistö A, Ramsey SA, Prinz S, Shmulevich I, et al. Dynamic analysis of MAPK signaling using a high-throughput microfluidic single-cell imaging platform. Proc Natl Acad Sci U S A 2009;106:3758–63.

[22] Deng Y, Finck A, Fan R. Single-cell omics analyses enabled by microchip technologies. Annu Rev Biomed Eng 2019;21:365–93.

[23] Ma C, Fan R, Ahmad H, Shi Q, Comin-Anduix B, Chodon T, et al. A clinical microchip for evaluation of single immune cells reveals high functional heterogeneity in phenotypically similar T cells. Nat Med 2011;17:738–43.

[24] Lu Y, Xue Q, Eisele MR, Sulistijo ES, Brower K, Han L, et al. Highly multiplexed profiling of single-cell effector functions reveals deep functional heterogeneity in response to pathogenic ligands. Proc Natl Acad Sci U S A 2015 17;112:E607–15.

[25] Xue Q, Lu Y, Eisele MR, Sulistijo ES, Khan N, Fan R, et al. Analysis of single-cell cytokine secretion reveals a role for paracrine signaling in coordinating macrophage responses to TLR4 stimulation. Sci Signal 2015;8:ra59.

[26] Kleppe M, Kwak M, Koppikar P, Riester M, Keller M, Bastian L, et al. JAK-STAT pathway activation in malignant and nonmalignant cells contributes to MPN pathogenesis and therapeutic response. Cancer Discov 2015;5:316–31.

[27] Xue Q, Bettini E, Paczkowski P, Ng C, Kaiser A, McConnell T, et al. Single-cell multiplexed cytokine profiling of CD19 CAR-T cells reveals a diverse landscape of polyfunctional antigen-specific response. J Immunother Cancer 2017;5:85.

[28] Rossi J, Paczkowski P, Shen YW, Morse K, Flynn B, Kaiser A, et al. Preinfusion polyfunctional anti-CD19 chimeric antigen receptor T cells are associated with clinical outcomes in NHL. Blood 2018;132:804–14.

[29] Kennedy E, Hokmabadi M, Dong Z, McKelvey K, Nelson EM, Timp G. Method for dynamically detecting secretions from single cells using a nanopore. Nano Lett 2018;18:4263–72.

[30] Chattopadhyay PK, Gierahn TM, Roederer M, Love JC. Single-cell technologies for monitoring immune systems. Nat Immunol 2014;15:128–35.

[31] Chen H, Ye F, Guo G. Revolutionizing immunology with single-cell RNA sequencing. Cell Mol Immunol 2019;16:242–9.

[32] Streets AM, Zhang X, Cao C, Pang Y, Wu X, Xiong L, et al. Microfluidic single-cell whole-transcriptome sequencing. Proc Natl Acad Sci U S A 2014;111:7048–53.

[33] Lichter P, Ledbetter SA, Ledbetter DH, Ward DC. Fluorescence in situ hybridization with Alu and L1 polymerase chain reaction probes for rapid characterization of human chromosomes in hybrid cell lines. Proc Natl Acad Sci U S A 1990;87:6634–8.

[34] Vanneste E, Melotte C, Debrock S, D'Hooghe T, Brems H, Fryns JP, et al. Preimplantation genetic diagnosis using fluorescent in situ hybridization for cancer predisposition syndromes caused by microdeletions. Hum Reprod 2009;24:1522–8

[35] Bengtsson M, Hemberg M, Rorsman P, Ståhlberg A. Quantification of mRNA in single cells and modelling of RT-qPCR induced noise. BMC Mol Biol 2008;9:63.

[36] Moignard V, Macaulay IC, Swiers G, Buettner F, Schütte J, Calero-Nieto FJ, et al. Characterization of transcriptional networks in blood stem and progenitor cells using high-throughput single-cell gene expression analysis. Nat Cell Biol 2013;15:363–72.

[37] Moignard V, Woodhouse S, Haghverdi L, Lilly AJ, Tanaka Y, Wilkinson AC, et al. Decoding the regulatory network of early blood development from single-cell gene expression measurements. Nat Biotechnol 2015;33:269–76.

[38] Xu L, Brito IL, Alm EJ, Blainey PC. Virtual microfluidics for digital quantification and single-cell sequencing. Nat Methods 2016;13:759–62.

[39] Chappell L, Russell AJC, Voet T. Single-cell (multi)omics technologies. Annu Rev Genomics Hum Genet 2018;19:15–41.

[40] Lawson DA, Kessenbrock K, Davis RT, Pervolarakis N, Werb Z. Tumour heterogeneity and metastasis at single-cell resolution. Nat Cell Biol 2018;20:1349–60.

[41] Ramsköld D, Luo S, Wang YC, Li R, Deng Q, Faridani OR, et al. Full-length mRNA-Seq from single-cell levels of RNA and individual circulating tumor cells. Nat Biotechnol 2012;30:777–82.

[42] Picelli S, Björklund ÅK, Faridani OR, Sagasser S, Winberg G, Sandberg R. Smart-seq2 for sensitive full-length transcriptome profiling in single cells. Nat Methods 2013;10:1096–8.

[43] Islam S, Kjällquist U, Moliner A, Zajac P, Fan JB, Lönnerberg P, et al. Characterization of the single-cell transcriptional landscape by highly multiplex RNA-seq. Genome Res 2011;21:1160–7.

[44] Hashimshony T, Wagner F, Sher N, Yanai I. CEL-Seq: single-cell RNA-Seq by multiplexed linear amplification. Cell Rep 2012;2:666–73.

[45] Jaitin DA, Kenigsberg E, Keren-Shaul H, Elefant N, Paul F, Zaretsky I, et al. Massively parallel single-cell RNA-Seq for marker-free decomposition of tissues into cell types. Science 2014;343:776–9.

[46] Hashimshony T, Senderovich N, Avital G, Klochendler A, de Leeuw Y, Anavy L, et al. CEL-Seq2: sensitive highly-multiplexed single-cell RNA-Seq. Genome Biol 2016;17:77.

[47] Klein AM, Mazutis L, Akartuna I, Tallapragada N, Veres A, Li V, et al. Droplet barcoding for single-cell transcriptomics applied to embryonic stem cells. Cell 2015;161:1187–201.

[48] Macosko EZ, Basu A, Satija R, Nemesh J, Shekhar K, Goldman M, et al. Highly parallel genome-wide expression profiling of individual cells using nanoliter droplets. Cell 2015;161:1202–14.

[49] Briggs JA, Weinreb C, Wagner DE, Megason S, Peshkin L, Kirschner MW, et al. The dynamics of gene expression in vertebrate embryogenesis at single-cell resolution. Science 2018;360:eaar5780.

[50] Wagner DE, Weinreb C, Collins ZM, Briggs JA, Megason SG, Klein AM. Single-cell mapping of gene expression landscapes and lineage in the zebrafish embryo. Science 2018;360:981–7.

[51] Plass M, Solana J, Wolf FA, Ayoub S, Misios A, Glažar P, et al. Cell type atlas and lineage tree of a whole complex animal by single-cell transcriptomics. Science 2018;360:eaaq1723.

[52] Papalexi E, Satija R. Single-cell RNA sequencing to explore immune cell heterogeneity. Nat Rev Immunol 2018;18:35–45.

[53] Gierahn TM, Wadsworth 2nd MH, Hughes TK, Bryson BD, Butler A, Satija R, et al. Seq-Well: portable, low-cost RNA sequencing of single cells at high throughput. Nat Methods 2017;14:395–8.

[54] Dura B, Choi JY, Zhang K, Damsky W, Thakral D, Bosenberg M, et al. scFTD-seq: freeze-thaw lysis based, portable approach toward highly distributed single-cell 3′ mRNA profiling. Nucleic Acids Res 2019;47:e16.

[55] Schaub A, Glasmacher E. Splicing in immune cells—mechanistic insights and emerging topics. Int Immunol 2017;29:173–181.

[56] Climente-González H, Porta-Pardo E, Godzik A, Eyras E. The functional impact of alternative splicing in cancer. Cell Rep 2017;20:2215–26.

[57] Brosseau JP, Lucier JF, Nwilati H, Thibault P, Garneau D, Gendron D, et al. Tumor microenvironment-associated modifications of alternative splicing. RNA 2014;20:189–201.

[58] Oltean S, Bates D. Hallmarks of alternative splicing in cancer. Oncogene 2014;33:5311–8.

[59] Sotillo E, Barrett DM, Black KL, Bagashev A, Oldridge D, Wu G, et al. Convergence of acquired mutations and alternative splicing of CD19 enables resistance to CART-19 immunotherapy. Cancer Discov 2015;5:1282–95.

[60] Xu MM, Pu Y, Zhang Y, Fu YX. The role of adaptive immunity in the efficacy of targeted cancer therapies. Trends Immunol 2016;37:141–53.

[61] Han A, Glanville J, Hansmann L, Davis MM. Linking T-cell receptor sequence to functional phenotype at the single-cell level. Nat Biotechnol 2014;32:684–92.

[62] Turchaninova MA, Britanova OV, Bolotin DA, Shugay M, Putintseva EV, Staroverov DB, et al. Pairing of T‐cell receptor chains via emulsion PCR. Eur J Immunol 2013;43:2507–15.

[63] DeKosky BJ, Kojima T, Rodin A, Charab W, Ippolito GC, Ellington AD, et al. In-depth determination and analysis of the human paired heavy- and light-chain antibody repertoire. Nat Med 2015;21:86–91.

[64] Busse CE, Czogiel I, Braun P, Arndt PF, Wardemann H. Single-cell based high-throughput sequencing of full-length immunoglobulin heavy and light chain genes. Eur J Immunol 2014;44:597–603.

[65] Stubbington MJT, Lönnberg T, Proserpio V, Clare S, Speak AO, Dougan G, et al. T cell fate and clonality inference from single-cell transcriptomes. Nat Methods 2016;13:329–32.

[66] Redmond D, Poran A, Elemento O. Single-cell TCRseq: paired recovery of entire T-cell alpha and beta chain transcripts in T-cell receptors from single-cell RNAseq. Genome Med 2016;8:80.

[67] Afik S, Yates KB, Bi K, Darko S, Godec J, Gerdemann U, et al. Targeted reconstruction of T cell receptor sequence from single cell RNA-seq links CDR3 length to T cell differentiation state. Nucleic Acids Res 2017;45:e148.

[68] Singh M, Al-Eryani G, Carswell S, Ferguson JM, Blackburn J, Barton K, et al. High-throughput targeted long-read single cell sequencing reveals the clonal and transcriptional landscape of lymphocytes. Nat Commun 2019;10:3120.

[69] Macaulay IC, Ponting CP, Voet T. Single-cell multiomics: multiple measurements from single cells. Trends Genet 2017;33:155–68.

[70] Packer J, Trapnell C. Single-cell multi-omics: an engine for new quantitative models of gene regulation. Trends Genet 2018;34:653–65.

[71] Hu Y, An Q, Sheu K, Trejo B, Fan S, Guo Y. Single cell multi-omics technology: methodology and application. Front Cell Dev Biol 2018;6:28.

[72] Han L, Zi X, Garmire LX, Wu Y, Weissman SM, Pan X, et al. Co-detection and sequencing of genes and transcripts from the same single cells facilitated by a microfluidics platform. Sci Rep 2014;4:6485.

[73] Dey SS, Kester L, Spanjaard B, Bienko M, van Oudenaarden A. Integrated genome and transcriptome sequencing of the same cell. Nat Biotechnol 2015;33:285–9.

[74] Macaulay IC, Haerty W, Kumar P, Li YI, Hu TX, Teng MJ, et al. G&T-seq: parallel sequencing of single-cell genomes and transcriptomes. Nat Methods 2015;12:519–22.

[75] Han KY, Kim KT, Joung JG, Son DS, Kim YJ, Jo A, et al. SIDR: simultaneous isolation and parallel sequencing of genomic DNA and total RNA from single cells. Genome Res 2018;28:75–87.

[76] Angermueller C, Clark SJ, Lee HJ, Macaulay IC, Teng MJ, Hu TX, et al. Parallel single-cell sequencing links transcriptional and epigenetic heterogeneity. Nat Methods 2016;13:229–32.

[77] Guo H, Zhu P, Wu X, Li X, Wen L, Tang F. Single-cell methylome landscapes of mouse embryonic stem cells and early embryos analyzed using reduced representation bisulfite sequencing. Genome Res 2013;23:2126–35.

[78] Hu Y, Huang K, An Q, Du G, Hu G, Xue J, et al. Simultaneous profiling of transcriptome and DNA methylome from a single cell. Genome Biol 2016;17:88.

[79] Hou Y, Guo H, Cao C, Li X, Hu B, Zhu P, et al. Single-cell triple omics sequencing reveals genetic, epigenetic, and transcriptomic heterogeneity in hepatocellular carcinomas. Cell Res 2016;26:304–19.

[80] Tang F, Barbacioru C, Nordman E, Li B, Xu N, Bashkirov VI, et al. RNA-Seq analysis to capture the transcriptome landscape of a single cell. Nat Protoc 2010;5:516–35.

[81] Clark SJ, Argelaguet R, Kapourani CA, Stubbs TM, Lee HJ, Alda-Catalinas C, et al. scNMT-seq enables joint profiling of chromatin accessibility DNA methylation and transcription in single cells. Nat Commun 2018;9:781.

[82] Liu L, Liu C, Quintero A, Wu L, Yuan Y, Wang M, et al. Deconvolution of single-cell multi-omics layers reveals regulatory heterogeneity. Nat Commun 2019;10:470.

[83] Darmanis S, Gallant CJ, Marinescu VD, Niklasson M, Segerman A, Flamourakis G, et al. Simultaneous multiplexed measurement of RNA and proteins in single cells. Cell Rep 2016;14:380–9.

[84] Genshaft AS, Li S, Gallant CJ, Darmanis S, Prakadan SM, Ziegler CGK, et al. Multiplexed, targeted profiling of single-cell proteomes and transcriptomes in a single reaction. Genome Biol 2016;17:18.

[85] Frei AP, Bava FA, Zunder ER, Hsieh EWY, Chen SY, Nolan GP, et al. Highly multiplexed simultaneous detection of RNAs and proteins in single cells. Nat Methods 2016;13:269–75.

[86] Stoeckius M, Hafemeister C, Stephenson W, Houck-Loomis B, Chattopadhyay PK, Swerdlow H, et al. Simultaneous epitope and transcriptome measurement in single cells. Nat Methods 2017;14:865–8.

[87] Peterson VM, Zhang KX, Kumar N, Wong J, Li L, Wilson DC, et al. Multiplexed quantification of proteins and transcripts in single cells. Nat Biotechnol 2017;35:936–9.
